# A framework for explaining the role of values in health policy decision-making in Latin America: a critical interpretive synthesis

**DOI:** 10.1186/s12961-020-00584-y

**Published:** 2020-09-07

**Authors:** C. Marcela Vélez, Michael G. Wilson, John N. Lavis, Julia Abelson, Ivan D. Florez

**Affiliations:** 1 McMaster Health Forum, McMaster University, 1280 Main Street West, Hamilton, ON L8S 4L6 Canada; 2 Department of Health Research Methods, Evidence and Impact, McMaster University, 1280 Main St. West, Hamilton, ON L8S 4K1 Canada; 3 Centre for Health Economics and Policy Analysis, McMaster University, 1280 Main St. West, Hamilton, ON L8S 4K1 Canada; 4 Health Policy PhD Program, McMaster University, 1280 Main St. West, Hamilton, ON L8S 4K1 Canada; 5 Department of Paediatrics, Faculty of Medicine, University of Antioquia, Cl. 67 #53 - 108, Medellín, Antioquia Colombia; 6 Department of Political Science, McMaster University, Hamilton, Canada; 7 Africa Centre for Evidence, University of Johannesburg, Johannesburg, South Africa

**Keywords:** Latin America, Critical interpretive synthesis, Values, Policy decision-making

## Abstract

**Background:**

Although values underpin the goals pursued in health systems, including how health systems benefit the population, it is often not clear how values are incorporated into policy decision-making about health systems. The challenge is to encompass social/citizen values, health system goals, and financial realities and to incorporate them into the policy-making process. This is a challenge for all health systems and of particular importance for Latin American (LA) countries. Our objective was to understand how and under what conditions societal values inform decisions about health system financing in LA countries.

**Methods:**

A critical interpretive synthesis approach was utilised for this work. We searched 17 databases in December 2016 to identify articles written in English, Spanish or Portuguese that focus on values that inform the policy process for health system financing in LA countries at the macro and meso levels. Two reviewers independently screened records and assessed them for inclusion. One researcher conceptually mapped the included articles, created structured summaries of key findings from each, and selected a purposive sample of articles to thematically synthesise the results across the domains of agenda-setting/prioritisation, policy development and implementation.

**Results:**

We identified 5925 references, included 199 papers, and synthesised 68 papers. We identified 116 values and developed a framework to explain how values have been used to inform policy decisions about financing in LA countries. This framework has four categories – (1) goal-related values (i.e. guiding principles of the health system); (2) technical values (those incorporated into the instruments adopted by policy-makers to ensure a sustainable and efficient health system); (3) governance values (those applied in the policy process to ensure a transparent and accountable process of decision-making); and (4) situational values (a broad category of values that represent competing strategies to make decisions in the health systems, their influence varying according to the four factors).

**Conclusions:**

It is an effort to consolidate and explain how different social values are considered and how they support policy decision-making about health system financing. This can help policy-makers to explicitly incorporate values into the policy process and understand how values are supporting the achievement of policy goals in health system financing.

**Trial registration:**

The protocol was registered with PROSPERO, ID=CRD42017057049.

## Background

Every health system in the world embodies values that guide health policy decisions [1–3]. Values, defined as “*principles, or criteria, for selecting what are good (or better, or best) among objects, actions, ways of life, and social and political institutions and structures*” [4], are essential at all stages of the policy process, ranging from the prioritisation of some issues over others on a government’s agenda to the development of policy options to address an issue and the implementation of selected policy options. When governments or institutions more generally set agendas and develop and implement policies, they also legitimise and promote certain values over others, making value-laden decisions about health systems [5].

Although values underpin the goals pursued in health systems and often the means for achieving them, including how health systems and particular health policies benefit the population, it is often not clear how values are incorporated into policy decision-making about health systems. This is perhaps not surprising given the complexity of decision-making about health systems, the wide range of values prioritised (and advocated for) by different stakeholders, and the many ways in which values can drive policy decisions as well as the reality that policy-makers often do not want to be explicit about the values used in the policy decision-making process [6]. Clarity may be all the more necessary in the resource-constrained health systems of low- and middle-income countries, where the values guiding how to get cost-effective treatments to those who need them and to achieve a better health status of their populations can have particular direct impacts [1].

Specifically in Latin America, the identification of values used in the policy decision-making process is an emerging field, with a paucity of evidence about the role of values and how they inform the prioritisation, development and implementation of policies in different contexts. Latin America has a vibrant history of political fluctuations – in the last 30 years, political contexts have spanned the spectrum from authoritarian governments to democracies led by right-, centre- or left-aligned governments. The region faces several challenges in the financing of its healthcare systems. The total health expenditure as a percentage of GDP ranges between 5% and 9%, with publicly funded health expenditure being below 6% for many countries in the region [7]. Despite these circumstances, there is little evidence about how this context influences the values chosen to guide policy decision-making about both health systems financing and arrangements. Indeed, in our preliminary search for this synthesis, to the best of our knowledge, no systematic review has specifically focused on values in Latin American health systems.

Given the paucity of synthesised evidence, we focused on understanding how and under what conditions societal values inform decisions about health system financing. Insights in this field could help policy-makers and stakeholders to understand how values are being incorporated into the policy decision-making process and, potentially, to make changes to better support the more explicit use of values in health systems policy-making in Latin America.

## Methodology

We used critical interpretive synthesis (CIS), a methodology oriented to theory building through combining the process of conducting a systematic review with the qualitative research inquiry. The CIS was selected for this review given its appropriateness to answer research questions that need to draw on a heterogeneous body of literature that is not particularly well developed or focused [8, 9], which is the case with the literature related to the use of values to inform the policy decision-making process about health-system financing in Latin American countries [10]. The CIS approach is based on analysing the perceptions and interpretations drawn from a wide range of relevant sources to develop a framework that explains the phenomenon being studied. Moreover, the CIS approach is not based on a prespecified design or quality of documents but rather on the relevance of papers to the theory [9].

For our CIS design, we used an explicit and structured search of the indexed literature followed by a more inductive purposive selection of papers from the pool of relevant documents to include in the analysis. We also adopted an iterative approach to refining the research question to carry out additional searches to fill conceptual gaps that emerged during the analysis [11]. The primary purpose of the synthesis was to explain how factors may influence the way in which policy-makers in Latin America use values to make decisions about financing in their health systems and under what conditions values come to be influential in the policy-making process.

### Literature search

In December 2016, we searched 17 databases to identify the relevant literature (Applied Social Sciences Index and Abstracts, CINAHL, Embase, HealthSTAR, Health Systems Evidence, International Political Science Abstracts, LEYES, LILACS, MEDLINE, PAIS International, ProQuest Political Science, PsycINFO, SciELO, Social Science Abstracts, Sociological Abstracts, Web of Science Core Collection from Thomson Reuters, and Worldwide Political Science Abstracts). Collectively, these databases index literature from a diverse range of subject domains, which allowed us to identify articles addressing a broad spectrum of situations in which values inform decision-making about health system financing.

The search strategy was comprised of a controlled vocabulary including the National Library of Medicines MeSH terms, EMTREE terms and keywords. In general, our search combined terms related to the region of interest (e.g. Latin America) with contextual or intervention terms related to the topic area (i.e. health-system financing and financial arrangements) and with terms related to the main area of interest (i.e. values). In addition, we searched the websites of WHO, Pan American Health Organization (PAHO) and World Bank to identify additional published and unpublished literature. Lastly, we conducted purposive searches to identify the literature to fill conceptual gaps that emerged during our inductive process of synthesis and analysis (e.g. to understand how policy-makers address values like right to health, equity or universality).

The search strategy was developed in consultation with an experienced librarian and then peer-reviewed using the PRESS checklist before being finalised (see Supplementary material 1 for the detailed search strategy).

### Selection criteria

We included all empirical and non-empirical articles, without date range limits, written in English, Portuguese or Spanish, that focus on what authors considered ‘values’, ‘principles’ or ‘goals’ of health-system financing. Those values must inform the policy process in Latin American countries at the macro (i.e. supranational, national and sub-national) and meso (i.e. administrative regions, healthcare organisations) levels but not at the micro level (i.e. clinical decision-making by health professionals).

### Reference reviewing and article selection

#### Step 1 – reviewing

One researcher (CMV) reviewed and assessed the titles and abstracts of all references captured by our search strategy to exclude those references that did not address the topic of interest or a Latin American country. Second, two researchers (CMV and IDF) assessed the titles and abstracts of the remaining references to classify them as potentially relevant or to exclude them; any disagreement at this stage was addressed by including the reference in the next step. Third, we retrieved the full text of all potentially relevant articles, which were then reviewed independently in duplicate by two researchers (CMV and IDF) to make a final assessment of whether they were relevant, and hence included in the sample frame from which we drew our purposive sample for the synthesis. Any disagreement at this stage was resolved by consensus. A table of studies excluded at this stage was prepared to document the reasons for exclusion (see Supplementary material 2 for details of articles excluded).

#### Step 2 – conceptual mapping and purposive sampling

We conceptually mapped the included papers using a structured form. The form included categories for document features and for variables of interest, including setting/country, research/non-research, value(s) addressed or discussed, government agenda-setting factors drawn from Kingdon’s framework [12], and policy development and implementation factors drawn from the 3I + E (institutions, interests, ideas and external factors) framework (see Supplementary material 3 for details of frameworks) [13].

We then used this mapping exercise to identify areas that were conceptually rich and areas where there appear to be conceptual gaps, which served to guide our selection of a purposive sample of relevant papers. The purposive sample was selected based on the following criteria: (1) articles were conceptually rich, defined as articles that addressed two or more factors included in the conceptual mapping and which describe and discuss policy decisions in depth; (2) articles captured a breadth of perspectives across different Latin American countries; and (3) articles that provided perspectives from different periods of time. The principal investigator (CMV) performed the conceptual mapping as well as the assessment of which papers are likely to offer important conceptual insights and the countries of focus to select a conceptually rich set of papers for inclusion in the analysis. However, all 207 papers were used to develop the framework.

### Data extraction

In addition to categorising the included articles, we extracted relevant data from all the full papers by developing a summary of key findings and conclusions related to our compass question and the frameworks used for the analysis. A log-book was kept by CMV, consisting of organised memos that document emerging themes used in the synthesis of findings phase.

### Synthesis of findings

To allow for an interpretive synthesis, we used qualitative methods to analyse and synthesise data from a purposively selected set of included studies. Although this synthesis included an element of aggregation (i.e. identifying those findings that recurred most frequently across included studies), the primary function of this synthesis was interpretation. To do this, we used a constant comparative method throughout our analysis to develop an explanatory framework of how and under what conditions Latin American countries use values to make decisions about health system financing, which allowed us to ensure that our framework was grounded in the data from the included papers. For the analysis, factors that influence or explain how values are used and under what conditions these values inform the agenda-setting, policy development and implementation process of health system financing were used as independent (explanatory) variables whereas the use of the value in the policy process was the dependent variable.

## Results

The electronic database search yielded 6481 published articles. After removing duplicates, 5925 articles remained for screening; from these, we excluded 5528 records due to a lack of relevance and duplicates. Of the 397 full-text articles screened, 199 met the inclusion criteria; 8 papers were purposively identified to fulfil gaps of our theoretical framework, yielding a total of 207 papers that were conceptually mapped (see Supplementary material 4 for details of all conceptually mapped articles, including whether they were later purposively sampled after full-text review).

**Fig. 1:**
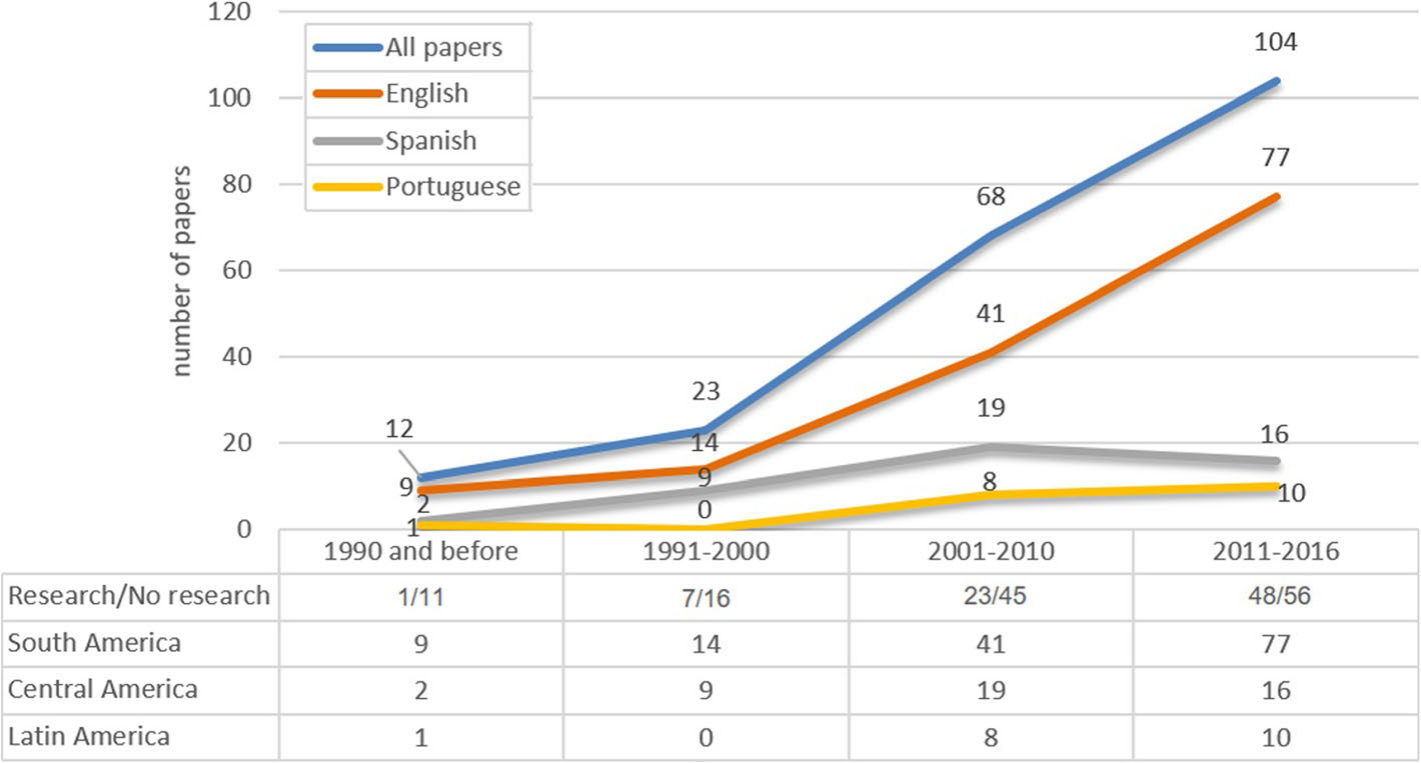
Characteristics of studies included by periods of time

**Fig. 2:**
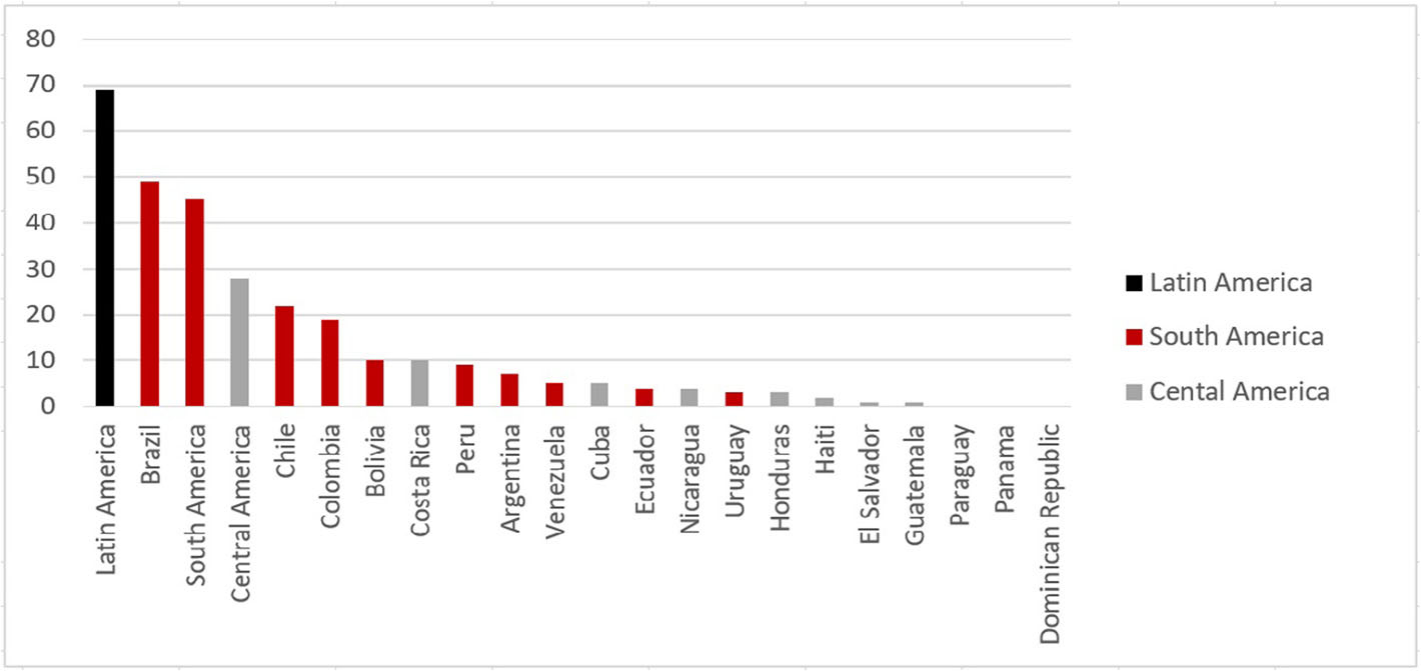
Number of times each country was specifically addressed

**Fig. 3:**
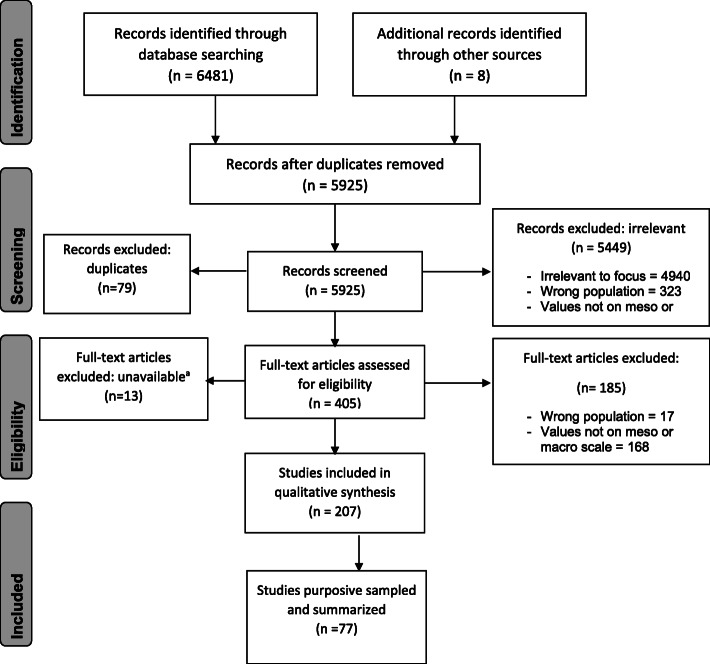
Prisma chart

Of the 207 included articles, 141 were written in English, 46 in Spanish and 19 in Portuguese. Articles focused on health systems of countries in South America (*n* = 84), Central America (*n* = 36), Central and South America (*n* = 14) or with a general scope in Latin America (*n* = 73) (Fig. 1). Brazil was the country most commonly addressed (22%; *n* = 45), followed by Mexico (14%; *n* = 28), Chile (11%; *n* = 22), Colombia (10%; *n* = 20) and Costa Rica (5%; *n* = 10) (Fig. 2). From the 79 articles that reported on findings from primary research, 12 were systematic reviews, 49 were quantitative studies, 26 were qualitative studies, and 4 used mixed methods [14–18]. The most common types of papers among the non-research articles were discussion papers (38%; *n* = 49) and situation analyses (23%; *n* = 30) (Table 1). From this list of 207 included articles, we selected 77 papers to include in our purposive sample for data synthesis (Fig. 3).

**Table 1:** Characteristics of all included papers and purposively sampled texts

	1990 and before		1991–2000		2001–2010		2011–2016	
	All papers included *n* = 12	Purposively sampled *n* = 6	All papers included *n* = 23	Purposively sampled *n* = 8	All papers included *n* = 68	Purposively sampled *n* = 25	All papers included *n* = 104	Purposively sampled *n* = 28
	*n* (%)	*n* (%)	*n* (%)	*n* (%)	*n* (%)	*n* (%)	*n* (%)	*n* (%)
Language								
English	9 (75)	4 (67)	14 (60)	6 (75)	41 (60)	14 (56)	77 (74)	22 (79)
Spanish	2 (17)	1 (17)	9 (40)	2 (25)	19 (28)	9 (36)	16 (15)	5 (18)
Portuguese	1 (8)	1 (17)	0	0	8 (12)	2 (8)	10 (10)	1 (4)
Region								
Central America	6 (50)	3 (50)	6 (26)	2 (25)	7 (11)	5 (20)	17 (16)	3 (11)
South America	2 (17)	1 (17)	4 (17)	1 (12)	26 (39)	7 (28)	52 (50)	15 (54)
Latin America	4 (33)	2 (33)	13 (57)	5 (63)	29 (43)	10 (10)	27 (26)	6 (21)
Central and South America	0	0	0	0	6 (9)	3 (12)	8 (8)	4 (14)
Primary research								
Yes	1 (8)	1 (17)	7 (30)	2 (25)	23 (34)	5 (20)	48 (46)	8 (29)
No	11 (92)	5 (83)	16 (70)	6 (75)	45 (66)	20 (80)	56 (54)	20 (71)
Type of research paper								
Quantitative	0	0	4 (17)	1 (12.5)	14 (21)	3 (12)	31 (30)	4 (14)
Qualitative	1 (8)	1 (17)	3 (13)	1 (12.5)	7 (10)	1 (4)	15 (14)	4 (14)
Mixed methods	0	0	0	0	2 (3)	1 (4)	2 (2)	0
Type of non-research papers								
Situation analysis	3 (25)	0	6 (26)	3 (37.5)	11 (16)	6 (24)	11 (11)	5 (18)
Discussion paper	5 (42)	4 (67)	5 (22)	1 (12.5)	20 (29)	11 (44)	19 (18)	9 (32)
Other	3 (25)	1 (17)	5 (22)	2 (25)	14 (21)	3 (12)	26 (25)	6 (21)

We identified 116 values in the 207 papers included (see Supplementary material 5 for the list and frequency of values identified). We found from this that stakeholders and policy-makers in Latin America call a great variety of things ‘values’ in their writings, including the right to health, equity, universality, sustainability, decentralisation, feasibility, privatisation, primary healthcare, Millennium Development Goals, and many more. Further, these values describe concepts that are quite different from each other, such as principles, strategies, instruments, specific goals, elements of a policy, or beliefs about the health system.

### Development of a framework

**Fig. 4:**
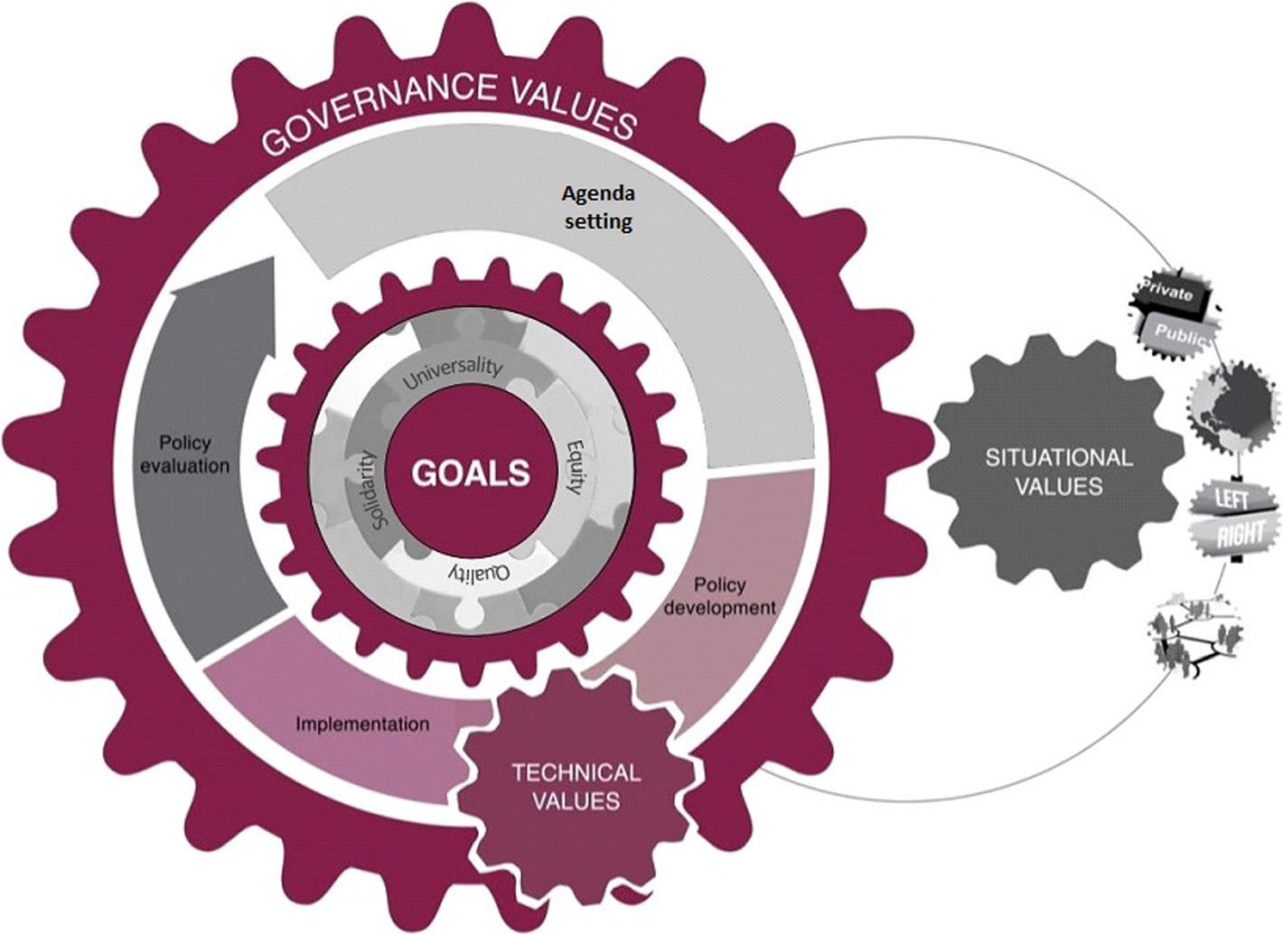
Graphical representation of the framework

We developed a framework to explain how values have been used to inform policy decisions about health system financing in Latin America (Table 2). The tension in developing such a framework is that values can be used as ends or means [6]. Therefore, values can be articulated as desirable outcomes to be achieved in the long term or used as concrete strategies or actions to achieve these desirable goals. Considering this, we have organised a framework according to four categories of values (Fig. 4) – (1) goal-related values (i.e. guiding principles that pursue the best healthcare for all and according to their needs, namely universality, equity, quality and solidarity); (2) technical values (i.e. those incorporated into the instruments and strategies adopted by decision-makers to ensure a sustainable and efficient health system); (3) governance values (i.e. those applied in the policy process to ensure a transparent and accountable process of decision-making); and (4) situational values (i.e. a broad category of values that represent competing strategies to make decisions in the health systems). We also identified four conditions under which situational values come to be influential in policy decision-making about health system financing in Latin America, namely (1) when aligned with policy legacies; (2) when aligned with the interests of influential groups; (3) when aligned with the ideology of the government; and (4) when aligned with international influences.

**Table 2:** Categories of values and how they are used

	What are they?	Values identified		How do they work
Goals	• Goal of health systems: the achievement of the best health for all according to their needs • Goals are classified as core values and intermediate values • Core values are equity, quality, solidarity and universality • Intermediate values are necessary factors to achieve final goals	Equity - Accessibility - Affordability - Afro-descendant equity - Availability - Cultural appropriateness - Fairness - Gender equity - Indigeneity - Protection of vulnerable population - Social justice Solidarity - Deservedness - Redistribution	Quality - Acceptability - Comprehensiveness - Continuity - Cultural appropriateness - Inclusiveness - Integrality - Reasonableness - Safety - Sufficiency - Timely access - User satisfaction Universality - Acceptability - Accessibility - Affordability - Availability - Equality - Free access - Gradualty - Progressiveness - Suitability - Utilisation	• Core values are guiding principles of health systems Each country in Latin America has prioritised some values to guide their health systems over others [19, 20]; for Costa Rica, they were equity, solidarity and universality; for Mexico, citizenship, fairness and solidarity; for Brazil, equity, participation and universality; for Chile, equity, participation and solidarity; for Colombia, solidarity and universality [21–25] • Intermediate values can be used like midway ends or like means to achieve core values For example, when talking about equity, we consider vertical and horizontal equity as well as accessibility, cultural appropriateness, fairness and gender equity. All these intermediate values not only serve to accomplish one of the core values, but they could also have intricate interrelationships to both help achieve more than one of the core values and to strengthen other intermediate values
Technical values	Principles that are incorporated into the instruments and strategies adopted by policy-makers to ensure that health-system goals are achieved rationally and informed by scientific evidence as well as the economic and social context	Efficiency related - Cost benefit - Cost effectiveness - Cost efficiency - Effectiveness - Efficacy - Efficiency - Financial protection - Sustainability	Rationale related - Austerity - Evidence based - Feasibility - Planning - Prioritisation - Professional autonomy - Rationality - Rationing	• Technical values are related to the instruments to achieve goals Used as strategies to ensure that the health system is able to deliver the best healthcare for all efficiently and sustainably “*The NHS* [Cuba] *is currently immersed in a thorough analysis of all health care levels with the intent of increasing effectiveness and efficiency, using limited resources to reconfigure services as necessary to achieve better patient-centered and population health outcomes.*” ([26], p. e18)
Governance values	Values of the political decision-making process that ensure the government considers the concerns of society and performs its functions in a transparent and accountable manner	Authority focused - Accountability - Enforcement of regulation - Governance - Responsiveness - Stewardship	Public focused - Public participation - Social participation - Transparency - Trust	• Governance values are related to the process of political decision-making Promote that health policies be developed and implemented with social legitimacy (i.e. policies are desirable, proper or appropriate within some socially constructed system of norms, values and beliefs) “*The concept of health governance refers to the way in which political actors within health system (providers) and the civil society (users, community leaders and NGOs), by means of explicit processes and rules, interact to produce, distribute and consume health as a good in relation to health services demand and population health needs*” ([27], p. 39)
Situational values	A broad category considering different factors that represent interests, ideas or visions of the health system, which vary according to changes in government or the social mood and that can strongly influence policy decision-making	Political system related - Hierarchisation - Reciprocity - Separation of functions - Sovereignty Health system structure related - Centralisation - Compulsoriness - Decentralisation - Intersectorality - Pluralism - Unification - Voluntariness Right to health oriented - Citizenship - Democratisation - Empowerment - Millennium Development Goals - Prevention - Primary healthcare - Public financing - Social cohesion	Management related - Institutional autonomy - Cost containment - Financial autonomy - Financial stability - Optimisation - Proportionality - Savings - Self-management - Simplicity - Transferability - Transparent procurement Delivery focused - Flexibility - Implementability - Mobility - Portability Market oriented - Competitiveness - Demand subsidies - Free choice - Individuality - Market - Privatisation - Profitability - Self-financing - Targeting	• Situational values come to be influential according to specific situational circumstances These situational factors depend on policy legacies, changes in the balance of organised forces, changes within the government, or international influences. Some situational values become crucial for a country at a specific time, and governments could incorporate them in the technical or governance categories, or even misrepresent their role and strongly pursue them as though they were a goal of the health system Many countries that implemented private health insurance models commonly asserted competitiveness, cost-containment, efficiency, market, privatisation and targeting as the most appropriate mechanisms to achieve universality in a liberal, market-oriented society [19–21, 25, 28–30]; those values are identified in the health system reforms of Brazil [21, 31–33], Chile [29, 32], Colombia [21, 32–34], Costa Rica [35] and Mexico [20, 28, 36, 37]; however, when we examined the strategies followed by more public financing-oriented governments, other values like public financing, primary healthcare and centralisation, appear

### How values are used

In describing these values, we provide interpretation (based on the literature identified from our searches) about how each is used to better understand their role in informing policy decisions (Table 2).

### Goal-related values

We developed this category with those values more commonly identified as guiding principles of health systems in the papers reviewed [21–25]. We made a distinction in this category between the core and intermediate values to be able to highlight that the core values of equity, quality, solidarity and universality, are those that best represent societal expectations for the health system. Additionally, these core values each contribute to the broader principle of the right to health, which is an important and ongoing matter of debate in Latin America (e.g. the increase of ‘tutelas’ in Colombia) [21, 38–40].

Despite core values being very important in health systems and commonly considered, they have different meanings or connotations depending on the perspective of each government. For example, universality is a value of almost every health system in Latin America [41] and a principle promoted by WHO [3, 42, 43]. However, while universality has been prioritised as a key goal, there is no consensus on its meaning and scope [29, 41, 44–46] and, therefore, it is not possible to design a common indicator to measure it or even agree on the extent to which it can be achieved [41] (Table 3).

**Table 3:** The conditions under which values are used in different stages of the policy process

Category & stage		Conditions under which values are used
Goals	Agenda setting	Problems • The lack of achievement of goals define the principal challenges faced by health systems ◦ Goals are problematized when recognized negatively (e.g., when framed as low quality, inequity, the lack of solidarity/universality, or vulnerability to the right to health) [23, 25, 27, 28, 47–50] • The perspective underlying the goals frames the problem in a specific way ◦ Although equity, quality, solidarity, and universality are important, commonly there is not a unique definition for each them [29, 51, 52]. In the case of equity, it is possible to identify egalitarian, utilitarian and “Rawlsian” approaches to equity. In an egalitarian perspective, health services should be distributed equally for all: in the utilitarian perspective, health services should be distributed based on who gets more out of them; and in a Rawlsian perspective, inequalities in health are allowed if the status of the disadvantaged people is better than in a scenario of complete equality [23, 53]
		Policies • The perspective underlying the goals shape how some issues gain prominence in the government agenda given that this is a precursor for identifying policy options. ◦ For example, there is no consensus on the meaning and scope of universality [29, 41, 44–46]. For legal and human right scholars, universality equates to the right to health and implies “equal or same entitlements” to the benefits of a health system [41]. From the perspective of health economists, universality is closely related to financial protection, which leads to a focus on policy options that prioritize prepaid mechanisms such as tax revenue, contributions from social health insurance, and private health insurance in order to minimize out-of-pocket payments and prevent financial bankruptcy. In contrast, from the perspective of public health, universality is considered in relation to defining population-level priorities in health, and the package of effective interventions that is needed to comprehensively address those needs [41]
		Politics • The comparison of the goal against what it has achieved is an important factor in agenda setting ◦ When governments compare their indicators of what the goal has achieved with their national expectations or indicators from other countries, a bad result could be a catalyst for agenda-setting [28, 47]
	Policy development and implementation	Institutions • Policies that are aligned with health-system goals are more likely to be prioritized for implementation ◦ This situation is especially the case when governments have signed on to international commitments such as the MDG [16, 54–56]
		Ideas • The perspective underlying the goals influence on what policy option is more likely to be chosen ◦ This determines how policies are developed and which policy options are more likely to pass [24, 32, 36, 44, 47, 57–59] • Policy alternatives that address intermediate goals may be preferred because they are more feasible to achieve ◦ Given that core values are very broad and imply the satisfaction of multiple dimensions, policies that focus on specific intermediate values might be preferred in policy development [60] • Goals are used as indicators for evaluating the general performance of the health system ◦ Indicators of equity, quality, solidarity, and universality are the most common ways to evaluate the global performance of health systems, even for policies that do not explicitly pursue the achievement of those goals [37] • Intermediate values are used as surrogate outcomes of evaluation of the performance of the health system ◦ Intermediate values are commonly used as dimensions or criteria to evaluate core values like equity or universality [61]
Technical values	Agenda setting	Problems • Technical values are used to frame problems regarding efficiency or financial sustainability ◦ Governments usually pay attention to problems that are framed in terms of inefficiency or menaces to the fiscal sustainability of the health systems [22–25, 28, 38, 62–64] • Technical values influence the government agenda when a problem puts the economic stability of a health system at risk ◦ One example is the accumulation of judiciary actions in the Colombian health system [65]
	Policy development and implementation	Institutions • Technical values are used as indicators of policy effectiveness, efficiency, and financial sustainability ◦ Sometimes, policymakers use indicators of financial protection to evaluate policies focused on achieving equity or universality [66, 67]
		Ideas • Technical values are used as pragmatic instruments to inform policy development ◦ For example, effectiveness and cost-effectiveness have been significant values to make decisions about what drugs or technologies are purchased or covered in Latin American health systems [45, 68–70] • Technical values are used to determine feasibility of implementing policies, and to prioritize those that are more feasible ◦ Goals such as universality are broad and complex; technical values help to find how to best achieve this goal by selecting policies that are technically possible and financially feasible [60, 71]
Governance values	Agenda setting	Problems • Governance values are used to frame problems in terms of corruption, failures in regulation or lack of social participation ◦ Recently, Latin American countries have come to frame problems of health systems in terms of corruption, lack of social participation or deficiencies in accountability [72]
		Policies • Governance values help to gain legitimacy in policy prioritization processes ◦ When social participation and other governance values are incorporated in the process of prioritization, governments can enhance the legitimacy of their initiatives [73]
	Policy development and implementation	Institutions
		• Governance values are typically used late in the process to improve the acceptability of the policy choice ◦ Social participation is often only considered when policies have been fully developed (e.g., for informing or notifying), and accountability is only considered by the governments as a report presented at the end of the year, which is not subject to auditing and feedback [74] • Governance values are used as strategies against corruption ◦ Transparency and accountability have begun to appear as essential values for policy implementation processes in health systems [25, 27, 28, 63, 75], and they are emphasized as strategies to prevent the corruption [33, 47, 76] • Governance values are used as indicators of good governance in the health system ◦ Good governance refers to how authority in the health system is exercised. Those values are used to monitor the performance of the government, and the engagement of the citizens in the policy process [27]
Situational values	Agenda setting	Problems • Situational values are used to frame problems according to specific situational influences ◦ Situational factors like the promulgation of international policies (e.g., MDGs), might influence how policymakers define problems to be consistent with the discourse of international agencies (e.g., paying attention to problems of maternal and child mortality) [30, 64, 77] • Situational values influence the government agenda when aligned international influences ◦ In the 1980s and 90s, “targeting” (i.e., establishing the basic minimum of health services by providing a subsidies with a preference to allocating them to low-income families) became prioritized as an important value to address problems of inequity, given that this value was aligned with the ideas promoted by the World Bank [21, 28, 32, 34, 51, 59].
	Policy development and implementation	Institutions • Situational values influence the policy selection when aligned with policy legacies ◦ Countries that implemented radical health system reforms during 80s and 90s, after intense political changes within the countries have not been able to introduce important transformations in the health system since then due to the strong resource, incentive, and interpretive effects that were created from the original reforms [63]
		Interests • Situational values influence what policy option is more likely to be chosen when aligned with interest of influential groups ◦ Policies that align with the interests or values of organized groups (e.g., doctors, patients, private sector) are more likely to be adopted [78] • Situational values are used as indicators of successful influence of specific groups or ideologies ◦ Situational values are used to evaluate the success of the government to implement their initiatives, the adoption of foreign policies (policy transfer), the power of some interest groups, and the level of progress in the implementation of a specific model of health system financing [79]
		Ideas • Situational values influence policy selection when aligned with the ideology of the government (e.g., left vs. right) ◦ When right-aligned governments prevail, generally health systems are influenced by values such as competitiveness, free choice, market, privatization, and targeting [19, 20, 51, 80]. In countries with left-aligned governments, values such as interculturality, public financing, prevention, and right to health prevail (e.g., Bolivia, Cuba, Ecuador, Venezuela) [26, 79, 81–84]
		External factors • Situational values influence the policy selection when aligned with international recommendations or requirements ◦ Latin America countries have been influenced by international agencies like the World Bank, the International Monetary Fund, WHO, PAHO, and UN. The influence of international agencies has been through a process of policy transfer, sometimes more persuasive and sometimes more coercive, which has resulted in many Latin American countries sharing a number of common characteristics [20, 25, 28, 29, 35, 45, 47, 57, 59, 81, 85–87] • Situational values are used to prioritize policies to be implemented when aligned with specific situational influences ◦ Perhaps the value that has had the greatest presence in the implementation of health reforms in Latin America has been decentralization, which was one of the key elements of the World Bank recommendations in the 80s and 90s [32, 47, 57, 88]

With regards to intermediate values, we propose that they are necessary factors in achieving goals. The role that intermediate values play in policy decision-making about health system financing varies based on the context of each country because they represent intermediate steps to achieving the goals of the health system. For example, Colombia and Costa Rica have declared universality as a guiding value of their health systems but, according to the perspective of each government, they have prioritised different intermediate values; for example, availability in Colombia and accessibility in Costa Rica [23].

### Technical values

Given that the right to health has deep and wide connotations and that the procedures, technologies, services and programmes that come to satisfy it are increasing in number and costs day by day, governments must define reasonable limits to ensure the achievement of goals. Those limits and the instruments to ensure that goal-related values are successfully reached should be defined by technical and rational rules that assure the efficiency of the resource allocation. In this regard, technical values like austerity, effectiveness, evidence, feasibility, planning, prioritisation, rationality or sustainability, are essential elements to the extent that they help to organise the health system to be durable over time. Sustainability is not by itself a final goal of the health system but a means for attaining health for all according to their needs.

### Governance values

The seriousness of health policy decision-making requires that the procedures for making decisions reflect values like accountability, social participation, stewardship and transparency. Governance values are not final goals of the health system but promote the principle of legitimacy in health policy development and implementation. For example, accountability and transparency have progressively begun to appear as essential values in Latin America [25, 27, 28, 63, 75] and they have been emphasised as imperative goals for addressing corruption [35, 47]. Governance values do not, on their own, achieve other goals or the materialisation of the right to health, but their presence in the policy decision-making process allows citizens to ensure that core values are considered in each policy decision.

### Situational values

Situational values are values that become important in specific circumstances. These values reflect policy legacies, changes in the balance of organised forces, interests of influential groups, ideological positions, changes in the national mood or international influences. We propose that situational values are not the ultimate goal of the health system. However, some situational values become tremendously important for a country at particular points in time and governments could incorporate them into technical or governance categories or even misrepresent their role and feverously pursue them as though they were a goal of the health system.

For example, at the end of the 80s and 90s, virtually all countries in Latin America began the process of reforming their health systems and pursued values promoted by the World Bank and the Inter-American Development Bank, including privatisation, competitiveness and market [20, 25, 28, 29, 45, 57, 85]. Reformers maintained that privatisation would improve other high standard values like accessibility, efficiency, equity, quality and social participation [35, 30, 47, 59, 81, 86, 87].

This situational values category is complex because different competing values belong here and there is no consensus about the legitimacy of those values. For example, some governments highlight decentralisation and others pursue centralisation; some countries promulgate compulsoriness and others ask for voluntariness; and some endorse public financing whereas others prefer privatisation. Countries that implemented private health insurance models commonly asserted competitiveness, privatisation, market, targeting, cost-containment and efficiency as the most appropriate mechanisms to achieve universality in a liberal, market-oriented society [19–21, 25, 28–30]. Those values are identified in the health system reforms of Brazil [21, 31–33], Chile [29, 32], Colombia [21, 32–34], Costa Rica [35] and Mexico [20, 28, 36, 37]. However, when we examined the strategies followed by more public financing-oriented governments, other values like public financing, primary healthcare and centralisation appeared (e.g. Bolivia, Cuba, Ecuador, Venezuela) [26, 81–84].

### The conditions under which values are used

Each category of values was analysed according to three stages of the policy process (i.e. agenda-setting, policy development and implementation) by considering the factors included in Kingdon’s agenda-setting framework and the 3I + E framework described in the methods section (Table 3).

### Agenda-setting

Values are used to frame problems in health systems and to prioritise issues on government agendas; they also shape how some issues gain prominence in the government agenda given that this is a precursor for identifying policy options.

When governments compare their indicators based on the goals they hope to achieve with similar indicators or expectations from other countries, such comparisons are important factors in framing an issue as one that warrants a government’s attention when those comparisons result in negatively framed goal-related values (e.g. lack of universality/solidarity, inequity, bad quality or vulnerability to the right to health) [23, 25, 27, 28, 47–50]. Governments also pay attention to problems that are framed in relation to inefficiency or as threats to the fiscal sustainability of health systems [22–25, 28, 38, 62, 63] and, more recently, to problems regarding corruption, lack of social participation or deficiencies in accountability.

Problems in health systems might also be defined in such a way that feedback from situational influences positions a specific strategy or value on the government agenda. Situational factors such as the promulgation of international policies (e.g. Millennium Development Goals) or the neoliberal reforms in the 80s and 90s might influence how policy-makers define problems to be consistent with the discourse of international agencies.

### Policy development

Policy alternatives that are aligned with the goals and perspectives of policy-makers are more likely to be considered and chosen. For example, a government addressing equity from a utilitarian perspective might be more interested in policies focused on providing financial protection to citizens than in policies focused on achieving gender equity [24, 29, 32, 37, 47, 57–59].

Technical values play an important role in policy development given that they are used as pragmatic instruments to develop and select policies that might be feasible and guarantee the sustainability of the health system. For example, effectiveness and cost-effectiveness have been significant values to make decisions about benefits plans, coverage of drugs or technologies, and development of clinical guidelines [45, 68, 69].

Governance values come to play a role because they are related to how elected officials and civil servants pay attention to societal groups’ demands for transparency and stewardship of the policy process. Policy-makers incorporate these values because they think it is the right way to make the decision-making process more efficient or because donors explicitly demand them [76]. We found that governance values are regularly used at the end of the policy development as a strategy to improve the social acceptability of the policy chosen. For example, many authors critique that governments are looking for social participation to reinforce the symbolic identification of health with democracy and not because they think that social participation would improve health system performance [86].

Situational values come to be influential when the policy option is aligned to (1) influential policy legacies (e.g. the financing structure of the health system, public versus private); (2) interests of influential groups; (3) the ideology of the government (e.g. left versus right); or (4) international recommendations or requirements.

As in others domains of the socio-political life in Latin America, values underlying the orientations of the current governing party (e.g. left versus right) are reflected in policy decision-making about health system financing (Table 3) [35, 88]. For example, when Brazil and Chile are followed over time, it is possible to identify values that represent the neoliberal ideology during the dictatorships and right-wing governments [33] but when those countries moved towards social-democratic governments, the prevailing values were right to health, equity, prevention and interculturality [33, 89].

Policies aligned with the values of international agencies/donors, their recommendations or requirements, are more likely to be chosen [30, 64, 90]. Several included papers highlight the role that the World Bank and the International Monetary Fund had in health systems reforms in Latin America and other low- and-middle-income countries worldwide. For example, the set of values promoted by these agencies in the 80s and 90s were adopted by Latin American countries by the diffusion of ideas or by coercion to access to loans [47, 59]. Other agencies, such as WHO, PAHO and the United Nations, have also influenced the set of values that are considered in decisions about health system financing. One of the mechanisms of influence is by national governments framing their health problems based on reports issued by these agencies [47, 48] and introducing and prioritising new values into the policy process based on these framings. The other mechanism is adopting strategies recommended by these international agencies to achieve those values [77, 91].

Despite the strong influence of international agencies over decision-making in Latin America, policy legacies are also important in the policy process and the values aligned with those legacies (e.g. the financing structure of the health system, public versus private) come to be influential. For example, while the World Bank promoted residual universalism, each Latin American country implemented policies to address universality in ways that were most aligned with the priorities of the governing party, policy legacies and the national mood. As a result, it is possible to identify a mix of traditional forms of universalism for workers and their dependents as well as minimal universalism for the unemployed, indigenous and vulnerable populations (e.g. Colombia) [29, 36, 57, 82].

Policies aligned with interests or the values of organised groups (doctors, patients, private sector) can also be influential. In some cases, influence can emerge from interest groups having strong connections to the government (e.g. pharmaceutical companies) and, in others, it can be driven by public opinion that is aligned with the interests of a group of patients or organisations/coalitions of doctors. Alternatively, influence can be driven by interest groups that are positioned in a way that can help to achieve other goals like accountability or help to assure the effectiveness of medical interventions. For example, the value of being evidence-based has been promoted by organisations of doctors and researchers, and it is an important technical value to ensure that health systems deliver cost-effective interventions and that decisions are made rationally.

### Policy implementation

Core goals-related values are the most common factors to evaluate the global performance of health systems, even for policies that do not explicitly pursue the achievement of those goals. Intermediate values are also considered as desirable and measurable surrogate outcomes of the health system and might be preferred by policy-makers because they are more attainable [16, 37, 54–56, 60].

Governments also consider technical values in the phase of policy implementation because values like efficacy, financial protection or sustainability can help them to achieve a better performance of the health system and help to gain accountability, transparency and trust in decision-making. The incorporation of new technical values, free of international pressures, is a phenomenon consistent with the development of technical capacities within the countries and the economic growth of Latin America.

Governance values have been used as indicators of how authority in the health system is exercised. Those values are used to monitor the performance of the government and the engagement of the citizens and interest groups into the policy process. Values such as accountability and transparency have progressively begun to appear as essential values for policy implementation [25, 27, 28, 63, 75] and are emphasised as imperative goals against corruption [33, 47].

Situational values are not only used to prioritise policies to be implemented when aligned with specific situational influences, but also for the evaluation of the successful implementation of particular initiatives of a government, the adoption of foreign policies (policy transfer), to extend the power of some interest groups and to measure the level of progress in the implementation of a specific model of health system financing. For example, decentralisation was a value frequently identified in the papers reviewed and, in fact, in the 80s and 90s many governments considered decentralisation as a goal of health systems [32, 47, 88]. Although decentralisation implied strategies that differed in scope, in the number of functions transferred, levels of government involved, and the participation or not of private organisations (i.e. deconcentration, delegation, devolution and privatisation) [88], this strategy was prioritised to be implemented for virtually all countries in the region.

## Discussion

### Principal findings

This review and the framework that emerged from the analysis are an effort to consolidate and explain how and under what conditions different social values are considered and how they support policy decision-making about health system financing in Latin America. We propose that the values considered in the policy process can be characterised in four ways, namely (1) goal-related values (i.e. guiding principles of the health system); (2) technical values (those incorporated into the instruments adopted by policy-makers to ensure a sustainable and efficient health system); (3) governance values (those applied in the policy process to ensure a transparent and accountable process of decision-making); and (4) situational values (a broad category of values that represent competing strategies to make decisions in the health systems). This theoretical framework is represented in Fig. 4, which can be thought of as a heuristic that can be used to identify the four categories of values and the conditions in which values are used in different stages of the policy process.

These categories of values come to be influential in government agenda-setting by framing the problems in specific ways, by prioritising some health issues in the government agenda, or by giving legitimacy to the process of agenda-setting [12]. In policy development, values are used as pragmatic instruments to inform policy development, influence what policy options are more likely to be chosen, and to improve the acceptability of the policy options that are selected [13]. In policy implementation, values influence which policies are more likely to be prioritised for implementation, used as indicators for evaluating the general performance of the health system, and used as indicators of good governance and as strategies against corruption.

We identified that values vary over the time, affected by different factors as governments and regimes change and as health systems reform. Our analysis of these variations is reflected on the framework we propose, with four conditions under which values influence policy decision-making about health system financing, namely (1) when aligned with policy legacies (e.g. the financing structure of the health system, public versus private); (2) when aligned with interests of influential groups; (3) when the policy option is aligned with government ideology (e.g. left versus right); and (4) when aligned with international recommendations or requirements.

In relation to the broader literature, we share two findings with the study of Giacomini et al. about values in the Canadian health system and we build on sets of values that have been outlined by international agencies. The first similar finding to Giacomini et al. is that stakeholders and policy-makers use ‘values’ to refer to many things, including different principles, strategies, instruments, specific goals, elements of a policy or beliefs about the health system. The second finding aligned with Giacomini et al. is that the contradictions about values are not between people for or against equity, but between people who prioritise equity and those who prioritise sustainability, or between people promoting policies addressing equity from an egalitarian approach and those who promote policies developed from a utilitarian or Rawlsian perspective [6].

In relation to core sets of values that have been previously articulated, our searches did not identify previous studies that developed a framework explaining the role of values in policy decision-making in Latin America, although WHO and the International Covenant on Economic, Social and Cultural Rights (ICESCR) have promoted the idea of a core set of values. These organisations prioritise values that guarantee “*the enjoyment of the highest attainable standard of physical and mental health*” [92]. The values (‘essential elements’) promoted by WHO and ICESCR are availability, accessibility, acceptability, quality and the delivery of healthcare free of any discrimination. Our framework shares the core value of quality but we propose that availability as well as accessibility and acceptability are components or intermediate steps to achieve the four most significant values needed to achieve the right to health.

There were two strengths and one potential limitation of this critical interpretive synthesis that are worth noting. The first strength is that the CIS was an appropriate methodological approach to synthesise heterogeneous sources of literature, empirical and conceptual papers considering or displaying debates about values in Latin American health systems, and articles discussing policies in health system financing. Second, the structured and systematic electronic search and the method of purposive sampling allowed us to be rigorous and transparent in the process of answering our research question.

One potential limitation of the study was that terms used in the literature were diverse and at times vague. Therefore, the search strategy may not have captured all the terms and concepts related to this topic. However, we performed a rigorous process of inclusion assessment independently of the papers identified in the searches in duplicate by two researchers to guarantee that different concepts, approaches and reflections of values were considered.

The results of our study are useful not only for policy-makers and stakeholders in Latin America but also for others in many countries that have implemented similar health system reforms. Both can use this framework to identify and understand how values have been and are being used in the process of prioritisation, policy development and implementation in light of the changing historical/political conditions. Additionally, policy-makers could use the framework to focus their policies according to their objectives (i.e. to achieve important goals, improve efficiency, gain legitimacy or respond to external influences). On the other hand, stakeholders interested in influencing policy agendas could use this framework to identify which values support or compete with the issues they want to prioritise and the policies that they think should be used to address them and/or how to make them more technically sound or socially supported.

The framework developed in this CIS can be used to analyse data or compare findings in future studies about the role of values in policy decision-making about health system financing in Latin American countries as well as in a different priority health policy domain (e.g. delivery arrangements or governance arrangements), in a different jurisdiction (e.g. developed countries, other low- and middle-income countries) or in a different policy field (e.g. education, child policy, social policy).

Future testing of this theoretical framework through case studies, cross-country comparisons or other methods that analyse specific financing decisions in Latin American health systems could be beneficial to identify gaps in the framework, additional mechanisms by which values are persuasive or other conditions under which values influence the policy process.

## Conclusions

The study of values in the policy decision-making process in Latin America is an emerging field. Our effort to synthesise current information and to develop a framework that explains their role in health system financing is a unique contribution to the body of knowledge in this field and provides an opportunity to explore the role of values in different policy decisions and jurisdictions.

## Supplementary information

**Additional file 1.** Literature search strategy.

**Additional file 2.** Articles excluded.

**Additional file 3.** Conceptual mapping according Kingdon’s Framework and 3Is Framework.

**Additional file 4.** Characteristics of all papers included.

**Additional file 5.** Values identified in papers reviewed.

## Data Availability

All data generated or analysed during this study are included in this published article and its supplementary information files.

## Abbreviations

3I + E: Institutions, interests, ideas and external factors
CIS: Critical interpretive synthesis
ICESCR: International Covenant on Economic, Social and Cultural Rights
PAHO: Pan-American Health Organization
