# Supplementary material for: A framework for explaining the role of values in health policy decision-making in Latin America: a critical interpretive synthesis
Source: Health Res Policy Syst. 2020 Sep 7;18:100. doi: 10.1186/s12961-020-00584-y (PMC7487839; doi:10.1186/s12961-020-00584-y)
Supplement: Supplementary file 1 — Additional file 1. Literature search strategy. [file 12961_2020_584_MOESM1_ESM.docx]

**Supplementary material 1. Literature search strategy**

| # | **Searches** |
| --- | --- |
| 1 | (Latin America or South America or Central America or low-income countries or middle-income countries or Argentin* or Bolivia* or Brazil* or Brasil* or Chile* or Colombia* or Costa Rica* or Cuba* or Ecuador or El Salvador or Guatemala or Haiti or Honduras or Mexic* or Nicaragua* or Panama or Paraguay* or Peru or Dominican Republic or Uruguay* or Venezuel*).af. |
| 2 | (((health adj system* adj financing).af. or financing.mp.) adj arrangement*.af.) or financing.af. or financia* adj en salud or financiamiento*.af.  [mp=title, original title, abstract, name of substance word, subject heading word] |
| 3 | (value* or principle* or goal*).af. or social values[MeSH Major Topic] or (valores* or principios* or valores sociales MeSH or metas*).af. |
| 4 | 1 and 2 and 3 |
