# Supplementary material for: A framework for explaining the role of values in health policy decision-making in Latin America: a critical interpretive synthesis
Source: Health Res Policy Syst. 2020 Sep 7;18:100. doi: 10.1186/s12961-020-00584-y (PMC7487839; doi:10.1186/s12961-020-00584-y)
Supplement: Supplementary file 2 — Additional file 2. Articles excluded. [file 12961_2020_584_MOESM2_ESM.docx]

**Supplementary material 2. Articles excluded**

**Values not on meso or macro scale**

| **#** | **Reference** |
| --- | --- |
| 1 | AHC MediaHIV/AIDS response differs among Latin American countries: some nations offer antiretrovirals; others cannot afford drug treatments. AIDS Alert. 2000;15(5):Online: https://www.ahcmedia.com/articles/58233-aids-alert-international-hiv-aids-response-differs-among-latin-american-countries. |
| 2 | Alkenbrack S, Chaitkin M, Zeng W, Couture T, Sharma S. Did equity of reproductive and maternal health service coverage increase during the MDG era? An analysis of trends and determinants across 74 low-and middle-income countries. PLoS ONE. 2015;10(9):e0134905. |
| 3 | Almeida G, Sarti FM. Measuring evolution of income-related inequalities in health and health care utilization in selected Latin American and Caribbean countries. Revista Panamericana de Salud Publica. 2013;33(2):83-89. |
| 4 | Alvarado-B E, Zunzunegui MV, Béland F, Sicotte M, Tellechea L. Social and gender inequalities in depressive symptoms among urban older adults of Latin America and the Caribbean. Journals of Gerontology Series B: Psychological Sciences & Social Sciences. 2007;62(4):S226-36. |
| 5 | Anderson I, Maliqi B, Axelson H, Ostergren M. How can health ministries present persuasive investment plans for women's, children's and adolescents' health? Bulletin of the World Health Organization. 2016;94(6):468-474. |
| 6 | Anonymous.In both Venezuela and Peru, 'simplified medicine' is goal. Hospital Practice. 1976;11(10):152-155-157. |
| 7 | Arza C. Pension Reforms in Latin America: Distributive Principles, New Inequalities, and Policy Options. Desarrollo Económico. 2009;49(195):363-388. |
| 8 | Asemota OA, Klatsky P. Access to infertility care in the developing world: the family promotion gap. Seminars in Reproductive Medicine. 2015;33(1):17-22. |
| 9 | Atun R, Knaul FM, Akachi Y, Frenk J. Innovative financing for health: what is truly innovative? Lancet. 2012;380(9858):2044-2049. |
| 10 | Baechler R. [The cost of primary health care. Administrative prices versus real costs]. Revista Medica De Chile. 1994;122(10):1207-1216. |
| 11 | Bahr J and Wehrhahn R. Life expectancy and infant mortality in Latin America. Social Science & Medicine. 1993;36(10):1373-1382. |
| 12 | Banke-Thomas AO, Madaj B, Charles A, van den Broek N. Social Return on Investment (SROI) methodology to account for value for money of public health interventions: a systematic review. BMC public health. 2015;15():582. |
| 13 | Bárcena A. Health protection as a citizen's right. Lancet. 2015;385(9975):e29-30. |
| 14 | Basu S, Stuckler D, McKee M. An alternative mechanism for international health aid: evaluating a Global Social Protection Fund. Health Policy and Planning. 2014;29(1):127-136. |
| 15 | Baulch B. Aid distribution and the MDGs. World Development. 2006;34(6):933-950. |
| 16 | Becerra-Posada F, de Snyder NS, Cuervo LG, Montorzi G. Priority research agendas: a strategic resource for health in Latin America. Revista Panamericana de Salud Publica. 2014;36(6):361-367. |
| 17 | Belizán JM, Cafferata ML, Belizán M, Althabe F. Health inequality in Latin America. Lancet. 2007;370(9599):1599-1600. |
| 18 | Bellows BW, Conlon CM, Higgs ES, Townsend JW, Nahed MG, Cavanaugh K, Grainger CG, Okal J, Gorter AC. A taxonomy and results from a comprehensive review of 28 maternal health voucher programmes. Journal of Health, Population, and Nutrition. 2013;31(4):106-128. |
| 19 | Bergonzoli G, Castellanos LG, Rodríguez R, Garcia LM. Determinants of tuberculosis in countries of Latin America and the Caribbean. Revista Panamericana de Salud Publica. 2016;39(2):101-105. |
| 20 | Bernardini-Zambrini DA. Healthy aging and intergenerational solidarity--Latin America and its moment of opportunity. Colombia Médica. 2012;43(2):112-113. |
| 21 | Biggs B, King L, Basu S, Stuckler D. Is wealthier always healthier? The impact of national income level, inequality, and poverty on public health in Latin America. Social Science & Medicine. 2010;71(2):266-273. |
| 22 | Biosca O, Brown H. Boosting health insurance coverage in developing countries: do conditional cash transfer programmes matter in Mexico? Health Policy and Planning. 2015;30(2):155-162. |
| 23 | Birn AE, Nervi L. Political roots of the struggle for health justice in Latin America. Lancet. 2015;385(9974):1174-1175. |
| 24 | Birn AE. Remaking international health: refreshing perspectives from Latin America. Revista Panamericana de Salud Publica. 2011;30(2):101-105. |
| 25 | Bittner MA, Dachs JNW, Tavares S, Soares LCR, Griesinger MO. Inequities in access to and use of drinking water services in Latin America and the Caribbean. Revista Panamericana de Salud Publica. 2002;11(5):386-396. |
| 26 | Boing AC, Bertoldi AD, Peres KG. Socioeconomic inequalities in expenditures and income committed to the purchase of medicines in Southern Brazil. Revista De Saude Publica. 2011;45(5):897-905. |
| 27 | Bonacim CA, de Araujo AM. [Evaluation of financial performance of health services: reflections of operational policies in the hospital sector]. Ciencia & Saude Coletiva. 2011;16():1055-1069. |
| 28 | Borghi J, Ensor T, Lissner C, Somanathan A, Craig L, Mills A, Lancet Maternal Survival Series steering groupMobilising financial resources for maternal health. Lancet. 2006;368(9545):1457-1465. |
| 29 | Brandão CM, Guerra AA Jr, Cherchiglia ML, Andrade EI, Almeida AM, da Silva GD, de Queiroz OV, Faleiros DR, Acurcio Fde A. [Expenses of the Brazilian Ministry of Health for high-cost drugs: a demographic and clinical analysis]. Value in Health. 2011;14(5):S71-77. |
| 30 | Burns JE, Mitrovich RC, Jauregui B, Matus CR, Andrus JK. Descriptive analysis of immunization policy decision making in the Americas. Revista Panamericana de Salud Publica. 2009;26(5):398-404. |
| 31 | Buss PM, Carvalho AI de. Health promotion in Brazil. Promotion & education. 2007;14(4):209-213. |
| 32 | Calikoglu S, Sapsin JW, Ruiz S, Mulreany JP. Water privatization and public health in Latin America. Revista Panamericana de Salud Publica. 2006;19(1):23-32. |
| 33 | Carcaba A, Ventura J, Gonzalez E. Value efficiency analysis of health systems: Does public financing play a role? Journal of Public Health. 2010;18(4):337-50. |
| 34 | Cardona-Valladares RD. Mapping HIV spending in Central America: Providing evidence for a renewed policy agenda. Journal of the International AIDS Society. 2012;15():216-218. |
| 35 | Carvalho MS, Schramm JMDA, Lima CRDA. Government funding for health and the reliability of national databases in Brazil, 2001-2002. Cadernos de SAúde Pública. 2006;22(9):1855-1864. |
| 36 | Casanovas JV, Rahmani-Ocora L, Samson M, Hogerzeil HV.Is access to essential medicines as part of the fulfilment of the right to health enforceable through the courts? Lancet. 2006;368(9532):305-311. |
| 37 | Casas SIC de. Geographical inequalities in mortality in Latin America. Social Science & Medicine. 1993;36(10):1349-1355. |
| 38 | Castillo-Laborde C, Silva-Illanes N. [Health technology assessment and its impact on pharmaceutical pricing and reimbursement policies]. Revista Medica De Chile. 2014;142():S33-38. |
| 39 | Cavagnero E, Evans DB, Lane C, Carrin G. Development assistance for health: should policy-makers worry about its macroeconomic impact? Bulletin of the World Health Organization. 2008;86(11):864-870. |
| 40 | Chernichovsky D, Martinez G, Aguilera N. Reforming "developing" health systems: Tanzania, Mexico, and the United States. Advances in Health Economics and Health Services Research. 2009;21():313-338. |
| 41 | Cid P C, Bastías S G. [Evaluation of financial status of public hospitals considering the updated costs of their services]. Revista Medica De Chile. 2014;142(2):161-167. |
| 42 | Cohen JC. Expanding drug access in Brazil: lessons for Latin America and Canada. Canadian Journal of Public Health. 2006;97(6):I-15. |
| 43 | Cortés R. The contemporary social policy debate in Latin America. Global Social Policy. 2008;8(1):109-114. |
| 44 | Costa WP. [National training systems]. Sistemas nacionais de treinamento. 1982;16(3):365-85. |
| 45 | Cotta RM, Machado JC. [The Bolsa Família cash transfer program and food and nutrition security in Brazil: a critical review of the literature]. Revista Panamericana de Salud Publica. 2013;33(1):54-60. |
| 46 | de Almeida JMC and Horvitz-Lennon M. Mental health care reforms in Latin America: An overview of mental health care reforms in Latin America and the Caribbean. Psychiatric Services. 2010;61(3):218-221. |
| 47 | Dettrick Z, Firth S1, Jimenez Soto E. Do strategies to improve quality of maternal and child health care in lower and middle income countries lead to improved outcomes? A review of the evidence. PLoS ONE. 2013;8(12):e83070. |
| 48 | do Socorro MQE. Avaliacao de efetividade de acoes de saneamento: analise de gestao. Ciencia & Saude Coletiva. 2005;10(2):493-497. |
| 49 | Dodd R and Piva P. Where did all the aid go? An in-depth analysis of increased health aid flows over the past 10 years. Bulletin of the World Health Organization. 2009;87(12):930-939. |
| 50 | El-Sadr WM, Gonsalves G, Mugyenyi P. No need for apologies. Journal of Acquired Immune Deficiency Syndromes. 2011;57():S68-71. |
| 51 | Escalante M, Gagliardino JJ, Guzmán JR, Tschiedel B. Call-to-action: timely and appropriate treatment for people with type 2 diabetes in Latin America. Diabetes Research & Clinical Practice. 2014;104(3):343-352. |
| 52 | Etienne CF. Achieving universal health coverage is a moral imperative. Lancet. 2015;385(9975):1271-1273. |
| 53 | Etienne CF. Equity in health systems. Revista Panamericana de Salud Publica. 2013;33(2):79-80. |
| 54 | Ewen M, Laing R, Ball D, Cameron A, Ross-Degnan D. Medicine prices, availability, and affordability in 36 developing and middle-income countries: a secondary analysis. Lancet. 2009;373(9659):240-249. |
| 55 | Fenwick TB. Stuck between the past and the future: Conditional cash transfer programme development and policy feedbacks in Brazil and Argentina. Global Social Policy. 2013;13(2):144-167. |
| 56 | Ferraz MB and Azevedo RT. Ministers of Health: short-term tenure for long-term goals? Sao Paulo Medical Journal. 2011;129(2):77-84. |
| 57 | Ferre JC. Economic Inequalities in Latin America at the Base of Adverse Health Indicators. International Journal of Health Services. 2016;46(3):501-522. |
| 58 | Filho AP. Inequities in access to information and inequities in health. Revista Panamericana de Salud Publica. 2002;11(5):409-412. |
| 59 | Franco-Giraldo A. Salud global: una visión latinoamericana. Revista Panamericana de Salud Pública. 2016;39(2):128-136. |
| 60 | Gandhi G, Lydon P, Cornejo S, Brenzel L, Wrobel S, Chang H. Projections of costs, financing, and additional resource requirements for low- and lower middle-income country immunization programs over the decade, 2011-2020. Vaccine. 2013;31():B137-148. |
| 61 | Garcia LP, Sant'Anna AC, Magalhães LC, Aurea AP. [Healthcare expenses of Brazilian families living in metropolitan areas: composition and trends during the period from 1995 to 2009]. Ciencia & Saude Coletiva. 2013;18(1):115-128. |
| 62 | Giuffrida A. Racial and ethnic disparities in Latin America and the Caribbean: a literature review. Diversity in Health & Care. 2010;7(2):115-128. |
| 63 | Gómez EJ. Wealth, Health, and Democracy in East Asia and Latin America. Journal of Health Politics, Policy & Law. 2011;36(2):353-356. |
| 64 | Gómez-Arias RD, Nieto E. Colombia: What has happened with its health reform? Revista Peruana de Medicina Experimental y Salud Pública. 2014;31(4):733-39. |
| 65 | Gonani A, Muula AS. The importance of Leadership towards universal health coverage in Low Income Countries. Malawi Medical Journal. 2015;27(1):34-37. |
| 66 | González E, Cárcaba A, Ventura J. Value efficiency analysis of health systems: Does public financing play a role? Journal of Public Health. 2010;18(4):337-350. |
| 67 | González-Block MÁ, Figueroa A, García-Téllez I, Alarcón J. Financial allocations in the System for Social Protection in Health in Mexico: challenges for strategic purchasing. Salud Publica De Mexico. 2016;58(5):522-532. |
| 68 | Grant K and Grant R. Health insurance and the poor in low income countries. World Hospitals and Health Services. 2003;39(1):19-22. |
| 69 | Grépin KA. Private Sector An Important But Not Dominant Provider Of Key Health Services In Low- And Middle-Income Countries. Health Affairs. 2016;35(7):1214-1221. |
| 70 | Hammonds, Rachel and Waris, Attiya and Criel, Bart and Van Damme, Wim and Whiteside, Alan and Ooms, Gorik Beyond health aid: would an international equalization scheme for universal health coverage serve the international collective interest? Globalization and Health. 2014;10():41. |
| 71 | Harris E. Financing social protection floors: Considerations of fiscal space. International Social Security Review. 2013;66(3):111-143. |
| 72 | Hausner H, Spiessl H, Hajak G. Abortion debate in Latin America and beyond…Lancet. 2007;370(9584):305-6. |
| 73 | Hertel-Fernández A. Retrenchment Reconsidered: Continuity and Change in the Post-authoritarian Institutions of Chilean Social Policy. Social Policy & Administration. 2009;43(4):382-396. |
| 74 | Hidalgo H, Chipulu M, Ojiako U. Risk segmentation in Chilean social health insurance. International Journal of Health Care Quality Assurance. 2013;26(7):666-681. |
| 75 | Hill PS, Buse K, Brolan CE, Ooms G. How can health remain central post-2015 in a sustainable development paradigm? Globalization and Health. 2014;10():18. |
| 76 | Holloway KA, Henry D. WHO essential medicines policies and use in developing and transitional countries: an analysis of reported policy implementation and medicines use surveys. PLoS medicine. 2014;11(9):e1001724. |
| 77 | Homedes UN. Corrigendum... Availability and affordability of new medicines in Latin American countries where pivotal clinical trials were conducted. Bulletin of the World Health Organization. 2016;94(5):404-404. |
| 78 | Horton R, Das P. Universal health coverage: not why, what, or when-but how? Lancet. 2015;385(9974):1156-1157. |
| 79 | Huish R. Going where no doctor has gone before: the role of Cuba's Latin American School of Medicine in meeting the needs of some of the world's most vulnerable populations. Public Health. 2008;122(6):552-557. |
| 80 | Johansson KA and Norheim OF. Problems with prioritization: exploring ethical solutions to inequalities in HIV care. The American journal of bioethics: AJOB. 2011;11(12):32-40. |
| 81 | Kaddar M and Furrer E. Are current debt relief initiatives an option for scaling up health financing in beneficiary countries? Bulletin of the World Health Organization. 2008;86(11):877-883. |
| 82 | Kilburn MR, Cannon JS. Home Visiting Start-Up: Lessons Learned From Program Replication in New Mexico. The journal of primary prevention. 2015;36(4):275-279. |
| 83 | Kouri G, Pelegrino JL, Munster BM, Guzmán MG. [Society, economy, inequities and dengue]. Revista Cubana De Medicina Tropical. 2007;59(3):177-185. |
| 84 | Krieger N. Latin American social medicine: the quest for social justice and public health. American Journal of Public Health. 2003;93(12):1989-1991. |
| 85 | Kuttner R. Guide to the GATT. New Republic. 1986;195(11):26-29. |
| 86 | Latko B, Temporão JG, Frenk J, Evans TG, Chen LC, Pablos-Mendez A, Lagomarsino G, de Ferranti D. The growing movement for universal health coverage. The Lancet. 2011;377(9784):2161-2163. |
| 87 | Leisinger KM, Garabedian LF, Wagner AK. Improving access to medicines in low and middle income countries: Corporate responsibilities in context. Southern Med Review. 2012;5(2):42802. |
| 88 | Levino A, Carvalho EF. Comparative analysis of health systems on the triple border between Brazil, Colombia, and Peru. Revista Panamericana de Salud Publica. 2011;30(5):490-500. |
| 89 | Lloyd-Sherlock P. Health sector reform in Argentina: a cautionary tale. Social Science & Medicine. 2005;60(8):1893-1903. |
| 90 | Lopez-Sarmiento A. [On the health sector crisis]. Correo Poblacional y de la Salud. 1997;5(3):7-9. |
| 91 | Maceira D, Paraje G, Aramayo F, Masi SD, Sánchez D. [Public financing of health research in five Latin American countries]. Revista Panamericana de Salud Publica. 2010;27(6):442-451. |
| 92 | Maimaris W, Paty J, Perel P, Legido-Quigley H, Balabanova D, Nieuwlaat R, McKee M. The Influence of Health Systems on Hypertension Awareness, Treatment, and Control: A Systematic Literature Review. Plos Medicine. 2013;10(7):e1001490. |
| 93 | Målqvist M. Abolishing inequity, a necessity for poverty reduction and the realisation of child mortality targets. Archives of Disease in Childhood. 2015;100():S5-9. |
| 94 | Mandigo M, O'Neill K, Mistry B, Mundy B, Millien C, Nazaire Y, Damuse R, Pierre C, Mugunga JC, Gillies R, Lucien F, Bertrand K, Luo E, Costas A, Greenberg SL, Meara JG, Kaplan R. A time-driven activity-based costing model to improve health-care resource use in Mirebalais, Haiti. Lancet. 2015;385():S22-S22. |
| 95 | Marin PP. [The situation of the elderly in Chile]. La situacion del adulto mayor en Chile. 1997;125(10):1207-12. |
| 96 | Martinez AD and Jimenez RE. Prevention of mental health in Mexico. Current state and perspectivesSalud Mental. 1999;22():154-158. |
| 97 | Mauch V, Bonsu F, Gyapong M, Awini E, Suarez P, Marcelino B, Melgen RE, Lönnroth K, Nhung NV, Hoa NB, Klinkenberg E. Free tuberculosis diagnosis and treatment are not enough: patient cost evidence from three continents. The International Journal of Tuberculosis and Lung Disease. 2013;17(3):381-387. |
| 98 | McKee M. Measuring the efficiency of health systems. British Medical Journal. 2001;323(7308):295-296. |
| 99 | Meiners-Chabin C. Financing free and universal access to antiretroviral drugs in the long-run: ART cost evolution in Brazil. Sexually Transmitted Infections. 2013;89():A42-A43. |
| 100 | Metaal P. Drug policy in the Americas Ã‡Â½Â¶?Â¶? a new set of Latin American policy proposals. Drugs & Alcohol Today. 2012;12(3):141-145. |
| 101 | Mtei G, Borghi J, Hanson K. Predicting Consumption Expenditure for the Analysis of Health Care Financing Equity in Low Income Countries: a Comparison of Approaches. Social Indicators Research. 2015;124(2):339-355. |
| 102 | Muntaner C, Rocha KB, Borrell C, Vallebuona C, Ibáñez C, Benach J, Sollar O. Social class and health in Latin America. Revista Panamericana de Salud Publica. 2012;31(2):166-175. |
| 103 | Murillo J, Prada-Trujillo G, Cahn P, Belloso WH. AIDS in Latin America. Infectious Disease Clinics. 2000;14(1):185-209. |
| 104 | Narayan R. The role of the People's Health Movement in putting the social determinants of health on the global agenda. Health Promotion Journal of Australia. 2006;17(3):186-188. |
| 105 | Nardi EP, Ferraz MB. Perception of the value of generic drugs in São Paulo, Brazil. Cadernos de Saúde Pública. 2016;32(2):e00038715. |
| 106 | Nascimento AC, Moysés ST, Werneck RI, Moysés SJ. Oral health in the context of primary care in Brazil. International Dental Journal. 2013;63(5):237-243. |
| 107 | Neal S, Channon AA, Carter S, Falkingham J. Universal health care and equity: evidence of maternal health based on an analysis of demographic and household survey data. International Journal for Equity in Health. 2015;14():56. |
| 108 | Nebot-Adell C, Rosales-Echevarria C, Borrell-Bentz RM. Development of primary health care competencies. Revista Panamericana de Salud Publica. 2009;26(2):176-183. |
| 109 | Neufeld LM, Gertler PJ, Fernald LCH. Role of cash in conditional cash transfer programmes for child health, growth, and development: an analysis of Mexico's Oportunidades. Lancet. 2008;371(9615):828-837. |
| 110 | Nieto E, López L, del Corral H, Marín D, Lopera LD, Benjumea D, Montes F, Molina G, Arbeláez MP. Cost-effectiveness of an alternative tuberculosis treatment: home-based guardian monitoring of patients. Revista Panamericana de Salud Publica. 2012;32(3):178-184. |
| 111 | Nishtar S. Health in the post-2015 agenda: three considerations in moving forward. Eastern Mediterranean Health Journal. 2014;20(2):71-72. |
| 112 | Oblitas FY, Loncharich N, Salazar ME, David HM, Silva I, Velásquez D. Nursing's role in tuberculosis control: a discussion from the perspective of equity. Revista Latino-Americana de Enfermagem (RLAE). 2010;18(1):130-138. |
| 113 | Oliveira MA and Chaves GC. A proposal for measuring the degree of public health-sensitivity of patent legislation in the context of the WTO TRIPS Agreement. Bulletin of the World Health Organization. 2007;85(1):49-56. |
| 114 | Ologunde R. The Challenges of Health System Financing. World Medical and Health Policy. 2013;5(4):403-411. |
| 115 | O'Neal JR. The affinity of foreign investors for authoritarian regimes. Political Research Quarterly. 1994;47(3):565. |
| 116 | Ooms G, Hammonds R, Waris A, Criel B, Van Damme W, Whiteside A. Beyond health aid: Would an international equalization scheme for universal health coverage serve the international collective interest? Globalization and Health. 2014;10(41):1-15. |
| 117 | Orpinas P, Ambrose A, Maddaleno M, Vulanovic L, Mejia M, Butrón B, Gutierrez GS, Soriano I. Lessons learned in evaluating the Familias Fuertes program in three countries in Latin America. Revista Panamericana de Salud Publica. 2014;36(6):383-390. |
| 118 | Osorio-de-Castro CG, Crisante M, Miranda ES, Oliveira EA, Oliveira MA. Proposed methodology for monitoring antiretroviral drugs price negotiations in Latin America and the Caribbean. Revista Panamericana de Salud Publica. 2009;26(2):137-147. |
| 119 | Paganini JM. Health services coverage in Latin America and the Caribbean. Revista Panamericana de Salud Publica. 1998;4(5):305-310. |
| 120 | Parada I, Arredondo A, Zúñiga A. Health care costs and financial consequences of epidemiological changes in chronic diseases in Latin America: evidence from Mexico. Public Health (Elsevier). 2005;119(8):711-720. |
| 121 | Paul E, Bodson O, Ridde V, Fecher F. La couverture santé universelle dans les pays à revenus faibles et intermédiaires : analyses économiques. Reflets et Perspectives de la Vie Economique. 2016;55(1):57. |
| 122 | Perreira KM, Telles EE. The color of health: Skin color, ethnoracial classification, and discrimination in the health of Latin Americans. Social Science & Medicine. 2014;116():241-250. |
| 123 | Pinheiro Filho FP, Sarti FM. Market and public policy network failures: challenges and possibilities for the Brazilian Unified Health System. Ciencia & Saude Coletiva. 2012;17(11):2981-2990. |
| 124 | Pinheiro Filho FP, Sarti FM. Market and public policy network failures: Challenges and possibilities for the Brazilian Unified Health System. Ciencia e Saude Coletiva. 2012;17(11):2981-2990. |
| 125 | Pinilla-Roncancio M. Disability and social protection in Latin American countries. Disability & Society. 2015;30(7):1005-1020. |
| 126 | Progress and inequity in Latin America. Lancet. 2007;370(9599):1589-1589. |
| 127 | Randall TC, Salicrup LA, Luciani S, Trimble EL. HPV Testing in Resource-Limited Settings: How Can We Reach the Next Level of Cervical Cancer Screening in Latin America and the Caribbean? Oncologist. 2015;20(10):1101-1104. |
| 128 | Reeves A, Gourtsoyannis Y, Basu S, McCoy D, McKee M, Stuckler D. Financing universal health coverage - Effects of alternative tax structures on public health systems: Cross-national modelling in 89 low-income and middle-income countries. The Lancet. 2015;386():274-80. |
| 129 | Reeves A, Gourtsoyannis Y, Basu S, McCoy D, McKee M, Stuckler D. Financing universal health coverage--effects of alternative tax structures on public health systems: cross-national modelling in 89 low-income and middle-income countries. Lancet. 2015;386(9990):274-280. |
| 130 | Reveiz L, Elias V, Terry RF, Alger J, Becerra-Posada F. Comparison of national health research priority-setting methods and characteristics in Latin America and the Caribbean, 2002-2012. Revista Panamericana de Salud Publica. 2013;34(1):42748. |
| 131 | Robert E, Ridde V. Global health actors no longer in favor of user fees: a documentary study. Globalization and Health. 2013;9():29. |
| 132 | Rocha PR, David HM. Determination or determinants? A debate based on the Theory on the Social Production of Health. Revista da Escola de Enfermagem da USP. 2015;49(1):127-133. |
| 133 | Roschke MA and Casas ME. [Formulation of a conceptual framework for continuing education in health]. Educacion Medica Y Salud. 1987;21(1):1-10. |
| 134 | Rudan I. Global health research priorities: mobilizing the developing world. Public Health. 2012;126(3):237-240. |
| 135 | Sachs JD. Primary health care in low-income countries: Building on recent achievements. JAMA. 2012;307(19):2031-2032. |
| 136 | Salazar L de. Effectiveness of health promotion and public health interventions: lessons from Latin American cases. Colombia Médica. 2010;41(1):85-97. |
| 137 | Sania N. Scaling up financing for health. Lancet. 2010;375(9719):983-983. |
| 138 | Santos AM, Giovanella L2. Regional governance: strategies and disputes in health region management. Revista De Saude Publica. 2014;48(4):622-631. |
| 139 | Santos-Mascarenhas R dos. Financing of the public health services. Revista de Saude Publica. 2006;40(4):559-572. |
| 140 | Schroeder P, Villen MT, Yen E, Crocco P. The Latin America and Caribbean Regional Health Sector Reform Initiative. Revista Panamericana de Salud Publica. 2000;8(1):135-139. |
| 141 | Schwarcz R and Fescina R. Maternal mortality in Latin America and the Caribbean. Lancet. 2000;356():S11-S11. |
| 142 | Servan-Mori E, Wirtz V, Avila-Burgos L, Heredia-Pi I. Antenatal Care Among Poor Women in Mexico in the Context of Universal Health Coverage. Maternal and Child Health Journal. 2015;19(10):2314-2322. |
| 143 | Siddiqi K and Newell J. What were the lessons learned from implementing clinical audit in Latin America? Clinical Governance. 2009;14(3):215-225. |
| 144 | Smith GR. Creating community power in health care. International Nursing Review. 1997;44(4):105-120. |
| 145 | Soares A. [Training and challenges for the health care system in Brazil: an analysis of investments to expand health care service delivery]. Cadernos de Saúde Pública. 2007;23(7):1565-1572. |
| 146 | Soares RR. Life expectancy and welfare in Latin America and the Caribbean. Health Economics. 2009;18():S37-54. |
| 147 | Sobel HL, Huntington D, Temmerman M. Quality at the centre of universal health coverage. Health Policy and Planning. 2016;31(4):547-549. |
| 148 | Souza-Noronha KVM de and Andrade-M V. Social inequality in health and the utilization of health services among the elderly in Latin America. Revista Panamericana de Salud Publica. 2005;17(5):410-418. |
| 149 | Strasser-Weippl K, Chavarri-Guerra Y, Villarreal-Garza C, Bychkovsky BL, Debiasi M, Liedke PE, Soto-Perez-de-Celis E, Dizon D, Cazap E, de Lima Lopes G Jr, Touya D, Nunes JS, St Louis J, Vail C, Bukowski A, Ramos-Elias P, Unger-Saldaña K, Brandao DF, Ferreyra ME, Luciani S, Nogueira-Rodrigues A, de Carvalho Calabrich AF, Del Carmen MG, Rauh-Hain JA, Schmeler K, Sala R, Goss PE.Progress and remaining challenges for cancer control in Latin America and the Caribbean. Lancet Oncology. 2015;16(14):1405-1438. |
| 150 | Stuckler D, Basu S, McKee M. Drivers of inequality in Millennium Development Goal progress: a statistical analysis. PLoS medicine. 2010;7(3):e1000241. |
| 151 | Teixeira JC, Gomes MH, Souza JA. [Association between sanitation services coverage and epidemiological indicators in Latin America: a study with secondary data]. Rev Panam Salud Publica. 2012;32(6):419-425. |
| 152 | Tellez TA and Aguayo TE Results of a private primary health system based on a family health care model in Chile. Revista Medica de Chile. 2008;136(7):873-879. |
| 153 | Templin T, Sadat N, Chapin A, Dieleman JL. Estimating future health spending by source in 184 countries, 2013-2040. The Lancet Global Health. 2016;4(Special issue):33. |
| 154 | The Lancet. Indigenous health in the Latin American golden decade. Lancet. 2016;387(10021):818-818. |
| 155 | Torres-Ruiz A. HIV/AIDS and sexual minorities in Mexico: a globalized struggle for the protection of human rights. Latin American Research Review. 2011;46(1):30-53. |
| 156 | Underwood J. The World Bank´s Response to the Developing Country Debt Crisis. Contemporary Policy Issues. 1989;7(2):50-65. |
| 157 | Vacca-González CP, Fitzgerald JF, Bermúdez JAZ. Defining generic drugs: an end or a means? Analysis of regulations in 14 countries in the Region of the Americas. Revista Panamericana de Salud Publica. 2006;20(5):314-323. |
| 158 | van der Putten IM, Evers SM, Deogaonkar R, Jit M, Hutubessy RC. Stakeholders' perception on including broader economic impact of vaccines in economic evaluations in low and middle income countries: a mixed methods study. BMC public health. 2015;15():356. |
| 159 | Vega J, Frenz P. Integrating social determinants of health in the universal health coverage monitoring framework. Revista Panamericana de Salud Publica. 2013;34(6):468-472. |
| 160 | Vlassoff M, Walker D, Shearer J, Newlands D, Singh S. Estimates of health care system costs of unsafe abortion in Africa and Latin America. International Perspectives on Sexual & Reproductive Health. 2009;35(3):114-121. |
| 161 | Voyi K. Is globalisation outpacing ethics and social responsibility in occupational health? La Medicina Del Lavoro. 2006;97(2):376-382. |
| 162 | Wagner AK, Quick JD, Ross-Degnan D. Quality use of medicines within universal health coverage: challenges and opportunities. BMC health services research. 2014;14():357. |
| 163 | Wagstaff A, Dmytraczenko T, Almeida G, Buisman L, Hoang-Vu Eozenou P, Bredenkamp C, Cercone JA, Diaz Y, Maceira D, Molina S, Paraje G, Ruiz F, Sarti F, Scott J, Valdivia M, Werneck H. Assessing Latin America's Progress Toward Achieving Universal Health Coverage. Health Affairs. 2015;34(10):1704-1712. |
| 164 | Walker AR, Glatthaar II, Labadarios D. Are our health goals realistic or idealistic? South African Medical Journal. 1995;85(4):235-237. |
| 165 | Weisbrot M. Changes in Latin America: consequences for human development. International Journal of Health Services. 2007;37(3):477-500. |
| 166 | Werner D.Health care and human dignity... community-based rural health programs in Latin America. Philippine Journal of Nursing. 1981;51():54-62. |
| 167 | Zakrison TL, Armada F, Rai N, Muntaner C. The politics of avoidable blindness in Latin America--surgery, solidarity, and solutions: the case of Misión Milagro. International Journal of Health Services. 2012;42(3):425-437. |
| 168 | Zeng W, Shepard DS, Chilingerian J, Avila-Figueroa C. How much can we gain from improved efficiency? An examination of performance of national HIV/AIDS programs and its determinants in low- and middle-income countries. Bmc Health Services Research. 2012;12():74. |

**Wrong population**

| **#** | **Reference** |
| --- | --- |
| 169 | Belka M and Robineau P. The MDGs in the European Region and Beyond: A Holistic Approach Needed to Correct Uneven Progress. UN Chronicle. 2008;45(1):35-36. |
| 170 | Bissell K, Perrin C, Beran D. Access to essential medicines to treat chronic respiratory disease in low-income countries. International Journal of Tuberculosis and Lung Disease. 2016;20(6):717-728. |
| 171 | Brindis CD, Newacheck PW, Halfon, N, Hughes DC. Improving children's access to health care: The role of decategorization. Bulletin of the New York Academy of Medicine. 1996;73(2):237-254. |
| 172 | Frenk J. Dimensions of health system reform. Health Policy. 1994;27(1):19-34. |
| 173 | Frenk J. The public-private Mix and Human-resources for Health. Health Policy and Planning. 1993;8(4):315-326. |
| 174 | Godal T. Opinion: do we have the architecture for health aid right? Increasing global aid effectiveness. Nature Reviews Microbiology. 2005;3(11):899-903. |
| 175 | Lundberg O, Åberg YM, Kölegård SM, Elstad JI, Ferrarini T, Kangas O, Norström T, Palme J Fritzell ,the NEWS Nordic Expert GroupThe role of welfare state principles and generosity in social policy programmes for public health: an international comparative study. Lancet. 2008;372(9650):1633-1640. |
| 176 | Mate KS, Rooney AL, Supachutikul A, Gyani G.Accreditation as a path to achieving universal quality health coverage. Globalization and Health. 2014;10():68. |
| 177 | Meda ZC, Konate L, Ouedraogo H, Sanou M, Hercot D, Sombie I. Leadership and vision in the improvement of universal health care coverage in low-income countries. Cahiers Sante. 2011;21(3):178-184. |
| 178 | Nijmeijer KJ, Fabbricotti IN, Huijsman R. Is franchising in health care valuable? A systematic review. Health Policy and Planning. 2014;29(2):164-176. |
| 179 | Peters DH, Paina L, Bennett S. Expecting the unexpected: applying the Develop-Distort Dilemma to maximize positive market impacts in health. Health Policy and Planning. 2012;27():44-53. |
| 180 | Sekabaraga C, Diop F, Soucat A. Can innovative health financing policies increase access to MDG-related services? Evidence from Rwanda. Health policy and planning. 2011;26():ii52-62. |
| 181 | Shaw D, Cook RJ. Applying human rights to improve access to reproductive health services. International Journal of Gynecology and Obstetrics. 2012;119(Suppl 1):S55-9. |
| 182 | Waitzkin H, Yager J, Santos R. Advancing the business creed? The framing of decisions about public sector managed care. Sociology of Health & Illness. 2012;34(1):31-48. |
| 183 | Walt G and Buse K. Aid coordination for health sector reform: a conceptual framework for analysis and assessment. Health Policy (Amsterdam, Netherlands). 1996;38(3):173-187. |
| 184 | Weaver JL. Reducing health care costs: responses of a multiethnic population. Inquiry. 1972;9(4):20-7. |
| 185 | Witter S, Fretheim A, Kessy FL, Lindahl AK. Paying for performance to improve the delivery of health interventions in low- and middle-income countries. Cochrane Database of Systematic Reviews. 2012;(2):84. |
