# Supplementary material for: A framework for explaining the role of values in health policy decision-making in Latin America: a critical interpretive synthesis
Source: Health Res Policy Syst. 2020 Sep 7;18:100. doi: 10.1186/s12961-020-00584-y (PMC7487839; doi:10.1186/s12961-020-00584-y)
Supplement: Supplementary file 3 — Additional file 3. Conceptual mapping according Kingdon’s Framework and 3Is Framework. [file 12961_2020_584_MOESM3_ESM.docx]

**Supplementary material 3. Conceptual mapping according Kingdon’s Framework and 3Is Framework**

| **Dependent variables** | **Independent variables** | **Data extraction question[s]** |
| --- | --- | --- |
| **Government agendas** [i.e., how do values play a role in determining which issues governments decide to take action on health system financing] | Problem | Explain whether and how the paper offers insights about the role of values into the prioritization of some problems related to the health system financing:   - focusing event; - change in an indicator; or - feedback from the operation of a current program or policy. |
|  | Policies/solutions | Explain whether and how the paper offers insights about the role of values into the selection of some policies/solutions over others to solve problems of health systems financing:   - diffusion of ideas; - feedback from the operation of an existing policy or program; - communication/persuasion;   If policies survived to the state of serious consideration explain:   - whether the policy viewed as technically feasible; - if the policy fits with the dominant values and current national mood; or - if it is acceptable in terms of current budget workability or likely political opposition or support. |
|  | Politics | Explain whether and how the paper offers insights about the role of values into the shaping of political factors that determine whether a government prioritize some issues of health system financing over others:   - swings in national mood; - change in the balance of organized forces; or - events within government |
| **Policy development and implementation** [i.e., how do values play a role in current policy decisions about health systems financing] | Institutions | Explain whether and how the paper offers insights about the role of values in shaping the structure of health system financing related to:   - government structures [e.g., federal versus unitary government]; - policy legacies [e.g., key past policies that facilitate and/or constrain future policy]; or - policy networks [e.g., executive council-appointed committees that involve a small number of key stakeholders vs. several arms-length interest groups each vying for the attention of political elites but with no formalized networks in place]. |
|  | Interests | Explain whether and how the paper offers insights about the role of values into what interest groups pursue or how they influence decisions about health system financing related to:   - types of interest groups that may be involved [e.g., societal interest groups, elected officials, civil servants or researchers]; - the specific interests in health system financing that each group may have; and - the influence/power each group might be able to wield. |
|  | Ideas | Explain whether and how the paper offers insights about the role of public values influencing decisions on health system financing related to:   - knowledge/beliefs about ‘what is’ [e.g., research knowledge]; and - views about ‘what ought to be’ [e.g., values]. |
| **Health system context** [cross-cutting variables] | Financial arrangements | Explain whether and how the paper offers insights about the role of values informing decisions related to:   - financing systems [i.e., mechanisms used to raise revenue for a particular health system]; - funding organizations [i.e., mechanisms used to pay for/purchase services from healthcare organizations within a health system]; - remunerating providers [i.e., mechanisms used to pay for/purchase services from, individual providers within a health system]; - purchasing products and services [i.e., mechanisms used to pay for/purchase products and services]; or - incentivizing consumers [i.e., financial or non-financial mechanisms to change specified behaviours of those who receive care]. |
|  | Governance arrangements | Explain whether and how the paper offers insights about the role of values informing decisions related to:   - policy authority (i.e., who makes policy decisions, how, using what types of frameworks, and on what terms); - organizational authority (i.e., who makes organizational decisions, how, using what types of frameworks, and on what terms); - commercial authority (i.e., who makes commercial decisions, how, using what types of frameworks, and on what terms); - professional authority (i.e., who makes professional decisions, how, using what types of frameworks, and on what terms); or - consumer & stakeholder involvement (i.e., how stakeholders are involved and on what terms). |
