# Supplementary material for: A framework for explaining the role of values in health policy decision-making in Latin America: a critical interpretive synthesis
Source: Health Res Policy Syst. 2020 Sep 7;18:100. doi: 10.1186/s12961-020-00584-y (PMC7487839; doi:10.1186/s12961-020-00584-y)
Supplement: Supplementary file 4 — Additional file 4. Characteristics of all papers included. [file 12961_2020_584_MOESM4_ESM.docx]

**Supplementary material 4. Characteristics of all papers included**

| **#** | **Reference** | **Language** | **Focus of the article** | **Countries** | **Primary research?** | **Type of paper** | **Values identified** | **Purposively sampled** |
| --- | --- | --- | --- | --- | --- | --- | --- | --- |
| [1] | J. L. García-Gutiérrez, “Health Planning in Latin America,” AJPH, vol. 65, no. 10, pp. 1047–1049, 1975. | English | History of health planning in Latin America | El Salvador, Argentina, Bolivia, Brazil, Chile, Colombia, Peru, Venezuela | No | Discussion paper | efficiency, planning | Y |
| [2] | J. A. Walsh and K. S. Warren, “Selective primary health care: an interim strategy for disease control in developing countries,” *Soc Sci Med*, vol. 14 C, pp. 145–163, 1980. | English | Primary health care and public health in developing countries | Latin America | No | Discussion paper | Accessibility, Cost-effectiveness, primary healthcare, planning, efficacy, feasibility | N |
| [3] | A. Ugalde, “Physicians’ control of the health sector: professional values and economic interests. Findings from the Honduran health system,” *Soc Sci Med*, vol. 14A, no. 5, pp. 435–444, 1980. | English | Physicians values, interests and control of the health system | Honduras | No | Discussion paper | planning, professional autonomy | Y |
| [4] | J. Castellanos Robayo and M. Kisil, “Estructura de los servicios de atención médica.,” *Educ Med Salud*, vol. 15, no. 3, pp. 258–290, 1981. | Spanish | Structure of health services | Latin America | No | Stakeholder input | universality, social justice, equity, efficiency, social participation, free choice, accessibility, rationing, quality | Y |
| [5] | L. C. G. Lobo, “Sistema de Saúde. Análise e propostas,” Educ Med Salud, vol. 20, no. 2, pp. 222–234, 1986. | Portuguese | Health systems analysis and proposals | Brazil | No | Discussion paper | universality, efficiency, equality, social participation, decentralization, intersectorality, rationing, quality, hierarchization | Y |
| [6] | M. C. Troncoso, S. Belmartino, C. Bloch, and I. Luppi, “El mercado de trabajo médico y la producción de servicios de salud en la Argentina,” Educ Med Salud, vol. 20, no. 4, pp. 535–558, 1986. | Spanish | The medical job market | Argentina | No | Theory paper | medical autonomy | N |
| [7] | P. Musgrove, “The economic crisis and its impact on health and health care in Latin America and the Caribbean,” Int. J. Health Serv., vol. 17, no. 3, pp. 411–441, 1987. | English | The economic crises and effects on health in Latin America | Latin America | No | Situation analysis | sustainability | N |
| [8] | J. L. Fiedler, “Recurrent cost and public health care delivery: the other war in El Salvador,” Soc. Sci. Med., vol. 25, no. 8, pp. 867–874, 1987. | English | Cost of health delivery | El Salvador | Yes | Case study | equity, efficiency, effectiveness, quality, simplicity, comfort | Y |
| [9] | L. M. Morgan, “Health without wealth? Costa Rica’s health system under economic crisis,” J. Public Health Policy, vol. 8, no. 1, pp. 86–105, 1987. | English | Effects of economic crisis on health system Costa Rica | Costa Rica | No | Situation analysis | universality, equality, equity, accessibility, "structured cross-class redistribution", primary healthcare, country sovereignty, austerity, market competition | N |
| [10] | R. M. Garfield, “War-related changes in health and health services in Nicaragua,” Soc. Sci. Med., vol. 28, no. 7, pp. 669–676, 1989. | English | Effects of the war on the health system in Nicaragua | Nicaragua | No | Situation analysis | Accessibility, availability | N |
| [11] | T. J. Bossert, “Can they get along without us? Sustainability of donor-supported health projects in Central America and Africa.,” Soc. Sci. Med., vol. 30, no. 9, pp. 1015–1023, 1990. | English | Sustainability of health programs after donor supports stop | Honduras, Nicaragua | No | Jurisdiction review | social participation, sustainability, effectiveness | N |
| [12] | L. M. Morgan, “International politics and primary health care in Costa Rica,” Soc. Sci. Med., vol. 30, no. 2, pp. 211–219, 1990. | English | Participation in primary health in Costa Rica | Costa Rica | No | Situation analysis | social participation, primary-care | Y |
| [13] | R. L. Robertson, C. E. Castro, L. C. Gómez, G. Gwynne, C. L. Tinajero Baca, and D. K. Zschock, “La Atención Primaria de Salud en el Ecuador: Los servicios del Ministerio de Salud y de la Seguridad Social Rural,” Bol Sanit Panam, vol. 111, no. 4, pp. 293–305, 1991. | Spanish | Financing of primary health care in Ecuador | Ecuador | Yes | Case study | equity, quality | N |
| [14] | C. Puentes-Markides, “Women and access to health care.,” Soc. Sci. Med., vol. 35, no. 4, pp. 619–626, 1992. | English | Women accessibility to health in Latin America | Latin America | No | Discussion analysis | equity, acceptability, accessibility, accountability, affordability, accommodation, gender equality | N |
| [15] | J. Frenk and M. A. Gonzalez-Block, “Primary care and reform of health systems: A framework for the analysis of Latin American experiences,” Heal. Serv. Manag. Res., vol. 5, no. 1, pp. 32–43, 1992. | English | Health reforms and primary health care in Latin America | Latin America | No | Framework | universality, efficiency, sustainability, decentralization, accessibility, primary-care, redistribution, integration, pluralism, quality, sustainability | Y |
| [16] | M. Vargas-Fuentes, “Privatización de servicios públicos. El caso de los servicios de salud en Costa Rica,” Salud Publica Mex., vol. 35, no. 2, pp. 186–193, 1993. | Spanish | Privatization of health services in Costa Rica | Costa Rica | No | Stakeholder position paper | universality, solidarity, social justice, equity, efficiency, social participation, decentralization, integrality, privatization, quality, compulsoriness | Y |
| [17] | H. Novaes, “Hospital trends in Latin America.,” World Health, vol. 47, no. 5, pp. 15–16, 1994. | English | Hospitals in the market of private insurance in Brazil | Brazil | No | Commentary | efficiency, privatization, quality | N |
| [18] | J. Frenk, “Comprehensive policy analysis for health system reform.,” Health Policy (New. York)., vol. 32, no. 1, pp. 257–277, 1995. | English | Health system reform in Mexico | Mexico | Yes | Case study | universality, solidarity, equity, efficiency, free choice, citizenship, pluralism, quality | Y |
| [19] | L. Lauwers, “Rawlsian equity and generalised utilitarianism with an infinite population ,” Econ. Theory, vol. 9, no. 1, pp. 143–150, 1996. | English | Rawlsian and utilitarian equity | Latin America | No | Theory paper | justice, equity | Y |
| [20] | E. Barillas, “La fragmentación de los sistemas nacionales de salud,” Rev Panam Salud Publica, vol. 1, no. 3, pp. 246–249, 1997. | Spanish | Fragmentation of health systems in LA | Latin America | No | Commentary | solidarity, equity, efficiency, decentralization, targeting | N |
| [21] | P. E. Brodwin, “Politics, practical logic, and primary health care in rural Haiti,” Med. Anthropol. Q., vol. 11, no. 1, pp. 69–88, 1997. | English | Primary health care in Haiti | Haiti | No | Commentary | social participation, primary healthcare, planning | N |
| [22] | F. Rojas-Ochoa and C. M. López-Pardo, “Economy, politics, and health status in Cuba,” Int. J. Heal. Serv., vol. 27, no. 4, pp. 791–807, 1997. | English | Financing decisions of the health system in Cuba | Cuba | No | Situation analysis | universality, efficiency, equality, decentralization, rationality, primary healthcare, planning | N |
| [23] | S. Russell and L. Gilson, “User fee policies to promote health service access for the poor: a wolf in sheep’s clothing?” Int. J. Heal. Serv., vol. 27, no. 2, pp. 359–379, 1997. | English | User fees in low- and middle-income countries | Latin America | Yes | Cross-sectional | equity, efficiency, decentralization, acceptability, accessibility, utilization, quality, transparency, acceptability, indigeneity, protection women and children | Y |
| [24] | J. M. Paganini, “La cobertura de la atención de salud en América Latina y el Caribe,” Rev. Panam. Salud Publica, vol. 4, no. 5, pp. 305–310, 1998. | Spanish | Health reforms and health coverage in LA | Latin America | Yes | Cross-sectional | equity, efficiency, accessibility, quality, primary healthcare | N |
| [25] | Y. Madrid, G. Velásquez, and E. Fefer, “The economics of pharmaceuticals and health sector reform in the Americas,” Pan Am J Public Heal., vol. 3, no. 5, pp. 343–350, 1998. | English | Pharmaceuticals and health reforms in Latin America | Latin America | No | Discussion paper | universality, solidarity, equity, efficiency, social participation, free choice, transparency, decentralization, availability, accessibility, liberalization, quality, affordability, rationality, cost-effectiveness | Y |
| [26] | AHRQ, “Managed care has spread from the United States to Latin America with an uncertain effect on quality and access to care.,” Agency for Healthcare Research and Quality, 1999. [Online]. Available: https://archive.ahrq.gov/research/jun99/ra20.htm#head1. | English | Impact of managed care on quality and access in LA | Latin America | No | Commentary | market | N |
| [27] | C. Vergara, “El contexto de las reformas del sector de la salud,” Rev. Panam. Salud Publica, vol. 8, no. 1, pp. 7–12, 2000. | Spanish | The context of health system reforms in LA | Latin America | No | Discussion paper | equity, efficiency, social participation, decentralization, competitiveness, targeting, autonomy | N |
| [28] | R. Molina, M. Pinto, P. Henderson, and C. Vieira, “Gasto y financiamiento en salud: situación y tendencias,” Rev. Panam. Salud Publica/Pan Am. J. Public Heal., vol. 8, no. 1, pp. 71–83, 2000. | Spanish | Health expenditure and financing in LA | Latin America | No | Situation analysis | equity, efficiency, sustainability, accessibility, privatization | N |
| [29] | I. M. Parada, P. Hernández, A. Arredondo, and F. Becerra, “Financiamiento de programas de farmacodependencia en la ciudad de México: 1990-1994,” Salud Publica Mex., vol. 42, no. 2, pp. 118–125, 2000. | Spanish | Financing of programs of drug abuse in Mexico | Mexico | Yes | Cross-sectional | efficiency, self-management, privatization | N |
| [30] | C. Celedón and M. Noé, “Reformas del sector de la salud y participación social,” Rev. Panam. Salud Publica, vol. 8, no. 1, pp. 99–104, 2000. | English | Social participation and health system reforms | Latin America | No | Discussion paper | equity, efficiency, social participation, free choice, timely access, efficacy, centralization, quality | N |
| [31] | A. Infante, I. de la Mata, and D. Lopez-Acuna, “Reforma de los sistemas de salud en America Latina y el Caribe: situacion y tendencias,” Rev Panam Salud Publica, vol. 8, no. 1, pp. 13–20, 2000. | Spanish | Health systems reforms in Latin America | Latin America | No | Situation analysis | equity, efficiency, social participation, sustainability, decentralization, effectiveness, quality | Y |
| [32] | T. J. Bossert et al., “Applied research on decentralization of health systems in Latin America: Colombia case study,” 2000. | English | Decentralization in LA | Bolivia, Chile | Yes | Comparative policy analysis | equity, efficiency, decentralization, accessibility, coverage, quality, financial soundness | N |
| [33] | J. L. Fiedler and J. B. Wight, “Financing health care at the local level: the community drug funds of Honduras,” Int J Heal. Mgmt, vol. 15, no. 4, pp. 319–340, 2000. | English | Financing drug funds at local level in Honduras | Honduras | Yes | Cross-sectional | social participation, accessibility, rationality | N |
| [34] | C. Collins, J. Araujo, and J. Barbosa, “Decentralising the health sector: issues in Brazil,” Health Policy (New. York)., vol. 52, no. 2, pp. 113–127, 2000. | English | Decentralization in Brazil | Brazil | No | Situation analysis | universality, equity, decentralization, public-financing | Y |
| [35] | A. Ross, J. Zeballos, and A. Infante, “La calidad y la reforma del sector de la salud en América Latina y el Caribe,” Rev. Panam. Salud Publica, vol. 8, no. 1, pp. 93–98, 2000. | Spanish | Quality and health system reform | Latin America | No | Discussion paper | equity, efficiency, social participation, sustainability, Quality, Effectiveness | N |
| [36] | G. González García, “Las reformas sanitarias y los modelos de gestión,” Rev. Panam. Salud Pública, vol. 9, no. 6, pp. 406–412, 2001. | Spanish | Health sector reform and management models | Latin America | No | Discussion paper | universality, equity, free choice, decentralization, autonomy, competitiveness, management, targeting, rationing, productivity | Y |
| [37] | M. Schlesser and B. Puertas, “Assessing community health among indigenous populations in Ecuador with a participatory approach: implications for health reform,” J. Community Health, vol. 26, no. 2, pp. 133–147, 2001. | English | Indigenous communities and health reform in Ecuador | Ecuador | Yes | Cross-sectional | equity, efficiency, protection of indigenous people, quality | N |
| [38] | C. Almeida, “Reforma de sistemas de servicios de salud y equidad en América Latina y el Caribe: algunas lecciones de los años 80 y 90,” Cad Saüde Pública, vol. 18, no. 4, pp. 905–925, 2002. | Spanish | Equity and health systems reform in Latin America | Latin America | No | Discussion paper | universality, solidarity, equity, equality, social participation, free choice, decentralization, cost-containment, right, management, competitiveness, market, efficacy, privatization, targeting | Y |
| [39] | S. M. Porto, “Equidad y distribución geográfica de recursos financieros en los sistemas de salud,” Cad. Saude Publica, vol. 18, no. 4, pp. 939–957, 2002. | Spanish | Equity and geographic distribution of financial resources in the Brazilian health system | Brazil | No | Performance review | universality, social justice, equity | N |
| [40] | N. Homedes and A. Ugalde, “Privatización de los servicios de salud: las experiencias de Chile y Costa Rica,” Gac Sanit, vol. 16, no. 1, pp. 54–62, 2002. | Spanish | Privatization of health services in Costa Rica and Chile | Costa Rica, Chile | Yes | Comparative situation analysis | solidarity, equity, efficiency, decentralization, privatization | N |
| [41] | I. Vargas, M. L. Vazquez, and E. Jane, “Equidad y reformas de los sistemas de salud en Latinoamerica,” Cad. Saude Publica, vol. 18, no. 4, pp. 927–937, 2002. | Spanish | Equity in health systems reform in Colombia and Costa Rica | Costa Rica, Colombia | No | Jurisdictional review | universality, solidarity, equity, efficiency, equality, free choice, efficacy, targeting, individualism | Y |
| [42] | Ó. Arteaga, S. Thollaug, A. C. Nogueira, and C. Darras, “Información para la equidad en salud en Chile,” Rev. Panam. Salud Publica/Pan Am. J. Public Heal., vol. 11, no. 5, pp. 374–385, 2002. | Spanish | Equity in health care in Chile | Chile | Yes | Cross-sectional | equity, decentralization | N |
| [43] | J. H. Bratt, M. A. Weaver, J. Foreit, T. de Vargas, and B. Janowitz, “The impact of price changes on demand for family planning and reproductive health services in Ecuador,” Health Policy Plan., vol. 17, no. 3, pp. 281–287, 2002. | English | Effects of prices change over reproductive services utilization in Ecuador | Ecuador | Yes | Randomized Controlled Trial | sustainability, accessibility, | N |
| [44] | R. A. Castano-Yepes, J. J. Arbelaez, U. B. Giedion, and L. G. Morales, “Equitable financing, out-of-pocket payments and the role of health care reform in Colombia,” Health Policy Plan., vol. 17, no. Suppl 1, pp. 5–11, 2002. | English | Equitable financing and out-of-pocket expenditure in Colombia | Colombia | Yes | Interrupted time series | equity, efficiency, fairness, progressiveness, regresiveness | N |
| [45] | G. Alleyne, “Equity and the goal of Health for All,” Rev. Panam. Salud Publica, vol. 11, no. 5/6, pp. 297–301, 2002. | English | Equity | Latin America | No | Commentary | equity, right | N |
| [46] | E. Gómez-Gómez, “Equidad de género en las políticas de reforma del sector de la salud de América Latina y el Caribe,” Rev. Panam. Salud Publica/Pan Am. J. Public Heal., vol. 11, no. 5–6, pp. 435–438, 2002. | Spanish | Equity and gender in the LA | Latin America | No | Government strategic plan for the health sector | Equity, gender equity | N |
| [47] | E. Gómez-Gómez, “Equidad, género y salud: retos para la acción,” Rev Panam Salud Publica, vol. 11, no. 5, pp. 435–438, 2002. | Spanish | Equity and gender in Latin America’s health systems | Latin America | No | Discussion paper | social justice, equity, social participation, gender-perspective | Y |
| [48] | R. M. Marques and A. Mendes, “A política de incentivos do Ministério da Saúde para a atenção básica: uma ameaça à autonomia dos gestores municipais e ao princípio da integralidade?,” Cad. Saude Publica, vol. 18, no. Supl, pp. 163–171, 2002. | Portuguese | Health financing in Brazil, conflicts between federal and municipal policies | Brazil | No | Situation analysis | universality, decentralization, integrality, Rationing, prioritization, right to health | N |
| [49] | J. Dachs, M. Ferrer, C. Florez, A. Barros, R. Narvá­ez, and M. Valdivia, “Inequalities in health in Latin America and the Caribbean: descriptive and exploratory results for self-reported health problems and health care in twelve countries.,” Rev. Panam. Salud Publica, vol. 11, no. 5/6, pp. 335–355, 2002. | English | Health inequalities in LA countries | Latin America | Yes | Cross-sectional | Equity, gender equity | N |
| [50] | M. Arretche, “Financiamento federal e gestão local de políticas sociais: o difícil equilíbrio entre regulação, responsabilidade e autonomia,” Cien. Saude Colet., vol. 8, no. 2, pp. 331–369, 2003. | Portuguese | Federal financing and local management in Brazil | Brazil | No | Situation analysis | universality, equity, efficiency, decentralization, enforcement of enforcement of regulation, accountability, autonomy, hierarchization | N |
| [51] | E. Rosselot, “Aspectos bioéticos comprendidos en la Reforma de la Atención de Salud en Chile. Los problemas del acceso y el costo de los recursos,” Rev. Med. Chil., vol. 131, no. 9, pp. 1079–1086, 2003. | Spanish | Bioethical problems of the health system reform in Chile | Chile | No | Discussion paper | universality, solidarity, social justice, equity, free choice, prevention, accessibility, timely access, effectiveness, financial-protection, primary-care, right, rationing | Y |
| [52] | A. C. Laurell, “What does Latin American social medicine do when it governs? The case of the Mexico City government,” Am. J. Public Health, vol. 93, no. 12, pp. 2028–2031, 2003. | English | The health policy of social medicine in Mexico | Mexico | No | Jurisdictional review | universality, solidarity, equity, decentralization, accessibility, timely access, privatization, market, targeting, cost-efficiency, right | Y |
| [53] | M. Romero-González, G. González, and R. A. Rosenheck, “Mental health service delivery following health system reform in Colombia,” J. Ment. Health Policy Econ., vol. 6, no. 4, pp. 189–194, 2003. | English | Mental health services with the health system reform in Colombia | Colombia | Yes | Before-after study | universality, efficiency, accessibility, managed competition, structured pluralism, financial autonomy, right to health, affordability | N |
| [54] | J. G. Temporão, “O mercado privado de vacinas no Brasil: a mercantilização no espaço da prevenção,” Cad. Saude Publica, vol. 19, no. 5, pp. 1323–1339, 2003. | Portuguese | The health policy of social medicine in Mexico | Brazil | Yes | Case study | universality, equity, equality, decentralization, accessibility, social-control, market | Y |
| [55] | N. Almeida-Filho, I. Kawachi, A. Filho, and J. Dachs, “Research on health inequalities in Latin America and the Caribbean: bibliometric analysis (1971-2000) and descriptive content analysis (1971-1995),” Am. J. Public Health, vol. 93, no. 12, pp. 2037–2043, 2003. | English | Health inequalities in LA | Latin America | Yes | Qualitative Systematic review | equity | N |
| [56] | L. M. Prada, “Aseguramiento en los regímenes Contributivo y Subsidiado, e impacto en los prestadores de servicios,” Rev. Salud Publica, vol. 6, no. 1, pp. 1–27, 2004. | Spanish | Effects of health insurance over providers in Colombia | Colombia | No | Performance review | efficiency | N |
| [57] | N. Palmer, D. H. Mueller, L. Gilson, A. Mills, and A. Haines, “Health financing to promote access in low income settings-how much do we know?,” Lancet, vol. 364, no. 9442, pp. 1365–1370, 2004. | English | Types and characteristics of health system financing in LMIC | Costa Rica, Honduras, Mexico, Brazil, Colombia | Yes | Quantitative systematic review | equity, efficiency, sustainability, accessibility, | N |
| [58] | S. Wallace and V. Gutiérrez, “Equity of access to health care for older adults in four major Latin American cities,” Rev. Panam. Salud Publica, vol. 17, no. 5/6, pp. 394–409, 2005. | English | Equity in health access for older people in LA | Mexico, Brazil, Chile, Uruguay | Yes | Cross-sectional | equity, availability, acceptability, accessibility | N |
| [59] | N. Homedes and A. Ugalde, “Why neoliberal health reforms have failed in Latin America.,” Health Policy (New. York)., vol. 71, no. 1, pp. 83–96, 2005. | English | Privatization and decentralization in Colombia and Chile | Chile, Colombia | No | Situation analysis | universality, equity, efficiency, decentralization, privatization, quality, user's satisfaction, accountability | Y |
| [60] | N. Homedes, A. Ugalde, and J. R. Forns, “The World Bank, pharmaceutical policies, and health reforms in Latin America,” Int. J. Heal. Serv. Planning, Adm. Eval., vol. 35, no. 4, pp. 691–717, 2005. | English | Pharmaceutical policies, world Bank and health systems reform in Latin America | Latin America | No | Discussion paper | decentralization, accessibility, affordability, privatization, targeting, cost-effectiveness, centralization, competitiveness, savings, adequate-use, transparency | Y |
| [61] | F. Knaul and J. Frenk, “Health insurance in Mexico: achieving universal coverage through structural reform,” Health Aff., vol. 24, no. 6, pp. 1467–1476, 2005. | English | Health insurance and universal coverage in Mexico | Mexico | No | Situation analysis | universality, solidarity, equity, efficiency, sustainability, quality, fairness, right, cost-effectiveness, consumer satisfaction, accountability | Y |
| [62] | F. Knaul, H. Arreolo-Ornelas, O. Mendez, and A. Martinez, “Justicia financiera y gastos catastróficos en salud: impacto del Seguro Popular de Salud en Mexico,” Salud Publica Mex., vol. 47, no. supl 1, pp. S54–S65, 2005. | Spanish | Financing and social insurance in Mexico | Mexico | Yes | Cross-sectional | universality, equity, efficiency, accessibility, fairness | Y |
| [63] | A. Ugalde and N. Homedes, “Las reformas neoliberales del sector de la salud: déficit gerencial y alienación del recurso humano en América Latina,” Rev Panam Salud Publica, vol. 17, no. 3, pp. 202–209, 2005. | Spanish | Human resources and health system reforms in LA | Latin America | No | Discussion paper | equity, efficiency, profitability, user-satisfaction, quality, flexibility | N |
| [64] | G. Carrin and C. James, “Key performance indicators for the implementation of social health insurance,” Appl. Health Econ. Health Policy, vol. 4, no. 1, pp. 15–22, 2005. | English | Performance Indicators for the Implementation of Social Health | Latin America | No | Framework | universality, equity, accessibility, affordability | N |
| [65] | A. Arredondo, E. Orozco, and E. De Icaza, “Evidences on weaknesses and strengths from health financing after decentralization: lessons from Latin American countries,” Int. J. Health Plann. Manage., vol. 20, no. 2, pp. 181–204, 2005. | English | Insurance in LMICs  health financing after decentralization in LA | Mexico, Nicaragua, Peru | Yes | Before-after study  Mixed methods | decentralization | Y |
| [66] | R. M. Marques and A. Mendes, “SUS e Seguridade Social: em busca do Elo Perdido,” Saude e Soc., vol. 14, no. 2, pp. 39–49, 2005. | Portuguese | Financing of social health insurance in Brazil | Brazil | No | Situation analysis | universality | N |
| [67] | D. Titelman and A. Uthoff, “The role of insurance in social protection in Latin America,” Int. Soc. Secur. Rev., vol. 58, no. 2–3, pp. 43–69, 2005. | English | Reforms in the financing of social security for healthcare and pensions in LA | Latin America | No | Situation analysis | universality, solidarity, equity, efficiency, accessibility, cost-containment, privatization | N |
| [68] | M. San Sebastián, A.-K. Hurtig, and K. Rasanathan, “Is trade liberalization of services the best strategy to achieve health-related millennium development goals in Latin America? A call for caution,” Rev. Panam. Salud Publica/Pan Am. J. Public Heal., vol. 20, no. 5, pp. 341–346, 2006. | English | Liberalization and MDG in LA | Latin America | No | Discussion paper | Millennium development goals | N |
| [69] | R. Urriola, “Chile: Protección social de la Salud,” Rev Panam Salud Publica, vol. 20, no. 4, pp. 273–286, 2006. | Spanish | Social protection in health in Chile | Chile | No | Discussion paper | universality, solidarity, equity, equality, social-cohesion, financial-protection, right, targeting, efficacy | Y |
| [70] | R. A. Montenegro and C. Stephens, “Indigenous health in Latin America and the Caribbean,” Lancet, vol. 367, no. 9525, pp. 1859–1869, 2006. | English | Indigenous communities and health policies in Latin America | Latin America | No | Situation analysis | accessibility, cultural appropriateness, appropriateness, autonomy | Y |
| [71] | C. Muntaner, R. M. Guerra-Salazar, S. Rueda, and F. Armada, “Challenging the neoliberal trend: the Venezuelan health care reform alternative.,” Can. J. Public Heal., vol. 97, no. 6, p. I-19, 2006. | English | Health system reform in Venezuela | Venezuela | No | Discussion paper | universality, solidarity, social justice, equity, social participation, intersectorality, prevention, accessibility, right, cultural-appropriateness, solidarity | Y |
| [72] | P. Lloyd-Sherlock, “When social health insurance goes wrong: Lessons from Argentina and Mexico,” Centro Interdisciplinario para el estudio de políticas públicas, Buenos Aires, 56, 2007. | English | Social health insurance in Argentina and Mexico | Mexico, Argentina | No | Situation analysis | solidarity, equity, sustainability, coverage, effectiveness, market, right | Y |
| [73] | C. Mesa-Lago, “Social Security in Latin America: Pension and Health Care Reforms in the Last Quarter Century,” Lat. Am. Res. Rev., vol. 42, no. 2, pp. 181–201, 2007. | English | Pensions and health care reform in Latin America | Bolivia, Argentina, Brazil, Colombia, Costa Rica, Mexico, Peru, Panama | No | Situation analysis | efficiency, social participation, free choice, decentralization, institutional autonomy, coverage, integration/coordination, competitiveness, privatization, efficiency | Y |
| [74] | C. O. Ocke-Reis, “Os desafios da ANS frente à concentração dos planos de saúde,” Cienc. e Saude Coletiva, vol. 12, no. 4, pp. 1041–1050, 2007. | Portuguese | Private health plan market in Brazil | Brazil | No | Discussion paper | Competitiveness, enforcement of enforcement of regulation, market | N |
| [75] | G. Cruces and D. Titelman, “Challenges for health and social protection in Latin America.,” Glob. Soc. Policy, vol. 7, no. 2, pp. 136–139, 2007. | English | Health and social protection in LA | Latin America | No | Commentary | universality, solidarity, efficiency, inclusiveness, rationality | N |
| [76] | C. Botto-Abella and B. Graterol-Mendoza, “Globalización, desigualdad y transmisión de las enfermedades tropicales en el Amazonas venezolano,” Cad. Saude Publica, vol. 23, no. Supl 1, pp. S51-63, 2007. | Spanish | Globalization, inequalities and Indigenous communities in Venezuela | Venezuela | No | Jurisdiction review | equity, decentralization, prevention, integrality, protection of indigenous people, right to health, public financing | N |
| [77] | F. Ruiz, L. Amaya, and S. Venegas, “Progressive segmented health insurance: Colombian health reform and access to health services,” Health Econ., vol. 16, no. 1, pp. 3–18, 2007. | English | Segmentation in health insurance in Colombia | Colombia | Yes | Cohort study | universality, solidarity, efficiency, decentralization, accessibility, utilization, coverage, targeting | Y |
| [78] | J. Raña K, J.-C. Ferrer O, and P. Bedregal G, “Modelo de asignación de recursos en atención primaria,” Rev. Med. Chil., vol. 135, no. 1, pp. 54–62, 2007. | Spanish | Model for health resource allocation for primary care in Chile | Chile | No | Framework | equity, efficiency, prevention, | N |
| [79] | J. J. S. P. Solla, A. A. C. dos Reis, A. P. M. Soter, A. S. Fernandes, and J. J. L. de Palma, “Mudanças recentes no financiamento federal do Sistema Único de Saúde: atenção básica à saúde,” Rev. Bras. Saude Matern. Infant., vol. 7, no. 4, pp. 495–502, 2007. | Portuguese | Public health system federal financing in Brazil | Brazil | No | Discussion paper | equity, decentralization | Y |
| [80] | M. E. Kruk, S. Galea, M. Prescott, and L. P. Freedman, “Health care financing and utilization of maternal health services in developing countries,” Health Policy Plan., vol. 22, pp. 303–310, 2007. | English | Health systems financing and access to maternal services in developing countries | Latin America | Yes | Cross-national analysis | Universality, MDG, effectiveness, utilization | Y |
| [81] | G. J. Schieber, P. Gottret, L. K. Fleisher, and A. A. Leive, “Financing global health: mission unaccomplished,” Health Aff. (Millwood)., vol. 26, no. 4, pp. 921–934, 2007. | English | Donors and health systems financing in poor countries | Latin America | No | Discussion paper | MDG, accountability | N |
| [82] | L. Tafur, “Controversia a la reforma de la Ley 100 de 1993, Ley 1122 de enero de 2007,” Colomb. Med., vol. 38, no. 2, pp. 107–112, 2007. | Spanish | Health system reform in Colombia (Law 1122) | Colombia | No | Discussion paper | universality, social participation, enforcement of enforcement of regulation | N |
| [83] | M. R. Bhatia and A. C. Gorter, “Improving access to reproductive and child health services in developing countries: are competitive voucher schemes an option?,” J. Int. Dev., vol. 19, no. 7, pp. 975–981, 2007. | English | Access to reproductive and child services and MDG in developing countries | Latin America | No | Discussion paper | efficiency, free choice, accessibility, MDG, private financing, quality, competitiveness, cost-effectiveness | N |
| [84] | I. Torres-Vigil, L. A. Aday, L. de Lima, and C. S. Cleeland, “Wath predicts the quality of advanced cancer care in Latin America? A look at five countries: Argentina, Brazil, Cuba, Mexico, and Peru.,” J. Pain Symptom Manag., vol. 34, no. 3, pp. 315–327, 2007. | English | Predictors of quality in cancer care in Latin America | Cuba, Mexico, Argentina, Brazil, Peru | Yes | Cross-sectional/Cross-national study | equity, availability, accessibility, quality, affordability | Y |
| [85] | A. Arredondo, O, and E. Orozco, “Equity, governance and financing after health care reform: lessons from Mexico,” Int. J. Health Plann. Manage., vol. 23, no. 1, pp. 37–49, 2008. | English | Equity, governance and health system financing in Mexico | Mexico | No | Discussion paper | equity, social participation, decentralization, accountability, democratization, governance | N |
| [86] | G. Backman et al., “Health systems and the right to health: an assessment of 194 countries.,” Lancet (London, England), vol. 372, no. 9655, pp. 2047–85, Dec. 2008. | English | Right to health global perspective | Latin America | Yes | Mixed methods | right to health | Y |
| [87] | A. L. D. Viana and C. V. Machado, “Proteção social em saúde: um balanço dos 20 anos do SUS,” Physis Rev. Saude Coletiva, vol. 18, no. 4, pp. 645–684, 2008. | Portuguese | Social protection in health in Brazil | Brazil | No | Discussion paper | decentralization, self-financing, private financing, right to health | N |
| [88] | M. del P. Guzmán-Urrea, “Deficiencia en los diagnósticos de las reformas sanitarias de los anos noventa en Améica Latina,” Rev Panam Salud Publica, vol. 25, no. 1, pp. 84–92, 2009. | Spanish | Equity and health systems reforms in Latin America | Latin America | No | Discussion paper | universality, equity, efficiency, decentralization, rationing, effectiveness, competitiveness, enforcement of enforcement of regulation | Y |
| [89] | J. Frenk, O. Gomez-Dantes, and F. M. Knaul, “The democratization of health in Mexico: financial innovations for universal coverage,” Bull. World Health Organ., vol. 87, no. 7, pp. 542–548, 2009. | English | Financial innovations and universal coverage in health in Mexico | Mexico | No | Situation analysis | universality, equity, sustainability, availability, democratization, utilization, financial-protection, quality, fair-financing, sufficiency, efficiency, right | Y |
| [90] | D. McIntyre, M. Thiede, and S. Birch, “Access as a policy-relevant concept in low- and middle-income countries,” Health Econ. Policy. Law, vol. 4, pp. 179–193, 2009. | English | Access concept in health systems | Latin America | No | Framework | availability, acceptability, accessibility, Utilization, affordability, empowerment | N |
| [91] | P. McNamee, J. Hussein, and L. Ternent, “Barriers in accessing maternal healthcare: evidence from low-and middle-income countries,” Expert Rev. Pharmacoecon. Outcomes Res., vol. 9, no. 1, pp. 41–48, 2009. | English | Allocation of expenditure, MDG and access to maternal and child care in LMICs | Latin America | Yes | Qualitative systematic review | availability, acceptability, MDG, utilization, cost-effectiveness, coverage, quality | N |
| [92] | A. D. Oxman and A. Fretheim, “Can paying for results help to achieve the Millennium Development Goals? A critical review of selected evaluations of results-based financing,” J. Evid. Based. Med., vol. 2, no. 3, pp. 184–195, 2009. | English | Pay-for-performance and MDG in LMICs | Latin America | Yes | Quantitative systematic review | MDG, effectiveness | N |
| [93] | M. Cintra, “Movimentação financeira: a base de uma contribuição para o INSS em substituição à folha de pagamentos,” RAP, vol. 44, no. 6, pp. 1477–1506, 2010. | Portuguese | Taxation and social security insurance in Brazil | Brazil | No | Situation analysis | Private financing | N |
| [94] | J. M. Lakin, “The End of Insurance? Mexico’s Seguro Popular, 2001 - 2007,” J. Health Polit. Policy Law, vol. 35, no. 3, pp. 313–352, 2010. | English | Health insurance in Mexico | Mexico | No | Discussion paper | equity, efficiency, competitiveness, coverage, pluralism, market, integration | Y |
| [95] | C. Méndez and J. Vanegas, “La participación social en salud: el desafío de Chile,” Rev Panam Salud Pública, vol. 27, no. 2, pp. 144–148, 2010. | Spanish | Social participation and health system reform in Chile | Chile | No | Discussion paper | solidarity, equity, efficiency, social participation, decentralization, accessibility, quality, market | N |
| [96] | O. Galárraga, S. G. Sosa-Rubí, A. Salinas-Rodríguez, and S. Sesma-Vásquez, “Health insurance for the poor: impact on catastrophic and out-of-pocket health expenditures in Mexico,” Eur J Heal. Econ, vol. 11, no. 5, pp. 437–447, 2010. | English | Health insurance and out-of-pocket expenditure in Mexico | Mexico | Yes | Cross-sectional | universality, financial protection, targeting | N |
| [97] | M. E. Kruk, D. Porignon, P. C. Rockers, and W. Van Lerberghe, “The contribution of primary care to health and health systems in low- and middle-income countries: a critical review of major primary care initiatives,” Soc. Sci. Med., vol. 70, no. 6, pp. 904–911, 2010. | English | Primary care and MDG in LMICs | Latin America | Yes | Qualitative systematic review | equity, efficiency, continuity, accessibility, MDG, primary-care, responsiveness, financial-protection, effectiveness, health-system-strengthening | N |
| [98] | R. Fryatt, A. Mills, and A. Nordstrom, “Financing of health systems to achieve the health Millennium Development Goals in low-income countries,” Lancet (London, England), vol. 375, no. 9712, pp. 419–426, 2010. | English | Health systems financing and MDG in LMICs | Latin America | No | Government strategic plan for the health sector | universality, accountability | Y |
| [99] | N. Krieger et al., “Who, and what, causes health inequities? Reflections on emerging debates from an exploratory Latin American/North American workshop.,” J. Epidemiol. Community Heal., vol. 64, no. 9, pp. 747–749, 2010. | English | Health inequalities in LA countries | Latin America | No | Stakeholder input | equality | N |
| [100] | C. F. Cáceres et al., “Implementation effects of GFATM-supported HIV/AIDS projects on the health sector, civil society and affected communities in Peru 2004-2007,” Glob. Public Health, vol. 5, no. 3, pp. 247–265, 2010. | English | Donations and health financing for AIDS, Malaria and TB in Peru | Peru | Yes | Case study | equity, accessibility, Accountability, financial-protection | N |
| [101] | S. Segall, “Is health (Really) Special? Health Policy between Rawlsian and Luck Egalitarian Justice,” J. Appl. Philos., vol. 27, no. 4, pp. 344–58, 2010. | English | Rawlsian and egalitarian justice | Latin America | No | Theory paper | justice, equity | Y |
| [102] | A. Stolkiner, “Derechos humanos y derecho a la salud en América Latina: la doble faz de una idea potente,” Med. Soc., vol. 5, no. 1, pp. 89–95, 2010. | Spanish | Right to health in LA | Latin America | No | Discussion paper | right to health | Y |
| [103] | I. Vargas, M. L. Vásquez, A. S. Mogollón-Pérez, and J.-P. Unger, “Barriers of access to care in a managed competition model: lessons from Colombia,” BMC Health Serv. Res., vol. 10, p. 297, 2010. | English | Managed care and access to health services in Colombia | Colombia | Yes | Case study | equity, efficiency, accessibility, Market, financial-protection, sustainability, profitability | N |
| [104] | T. M. Gonçalves Menicucci, “A Política de Saúde no Governo Lula,” Saude E Soc., vol. 20, no. 2, pp. 522–532, 2011. | Portuguese | The health policy in Brazil | Brazil | No | Government position paper | efficiency, integrality, accessibility, coverage, quality, focalization, private, gender-equity | N |
| [105] | J. R. M. de França and N. do R. Costa, “A dinâmica da vinculação de recursos para a saúde no Brasil: 1995 a 2004,” Cienc. e Saude Coletiva, vol. 16, no. 1, pp. 241–257, 2011. | Portuguese | Binding resources for financing health in Brazil | Brazil | No | Performance review | efficiency, sustainability, coverage, sufficiency | N |
| [106] | G. Z. Portela and J. M. Ribeiro, “A sustentabilidade econômico-financeira da Estratégia Saúde da Família em municípios de grande porte,” Cienc. e Saude Coletiva, vol. 16, no. 3, pp. 1719–1732, 2011. | Portuguese | Financial sustainability of the Family Health Strategy in Brazil | Brazil | Yes | Cohort study | universality, sustainability | N |
| [107] | A. C. G. do E. Santo and O. Y. Tanaka, “Financiamento, gasto e oferta de serviços de saúde em grandes centros urbanos do estado de São Paulo (Brasil),” Cien. Saude Colet., vol. 16, no. 3, pp. 1875–1885, 2011. | Portuguese | Health care financing and expenditure in Brazil | Brazil | Yes | Cohort study | equity, decentralization | N |
| [108] | L. Huamán-Angulo, L. Liendo-Lucano, and M. Núñez-Vergara, “Plansalud: Plan sectorial concertado descentralizado para el desarrollo de capacidades en salud, Perú 2010-2014,” Rev Peru Med Exp Salud Publica, vol. 28, no. 2, pp. 362–371, 2011. | Spanish | Descentralization and health plan for Peru | Peru | No | Government strategic plan for the health sector | equity, efficiency, decentralization, accessibility, quality, multiculturalism, relevance, | N |
| [109] | R. Uauy, “The impact of the Brazil experience in Latin America.,” Lancet, vol. 377, no. 9782, pp. 1984–1986, 2011. | English | Brazilian health system experience | Brazil | No | Commentary | universality, accessibility, effectiveness | N |
| [110] | K. Artaraz, “New Latin American networks of solidarity? ALBA’s contribution to Bolivia’s National Development Plan (2006-10),” Glob. Soc. Policy, vol. 11, no. 1, pp. 88–105, 2011. | English | Solidarity networks and health policy in Bolivia | Bolivia | No | Stakeholder input | universality, solidarity, social justice, fairness | Y |
| [111] | N. B. Sugiyama, “The diffusion of Conditional Cash Transfer programs in the Americas.,” Glob. Soc. Policy, vol. 11, no. 2–3, pp. 250–278, 2011. | English | Conditional cash transfer, policy diffusion in the Americas | Latin America | No | Situation analysis | efficiency, effectiveness | N |
| [112] | F. Augustovski, S. Garcia Marti, A. Pichon Riviere, and A. Rubinstein, “Universal coverage with rising healthcare costs; health outcomes research value in decision-making in Latin America,” Expert Rev. Pharmacoeconomics Outcomes Res, vol. 11, no. 6, pp. 657–659, 2011. | English | Universal coverage with healthcare costs rising in Latin America | Latin America | No | Stakeholder input | universality, efficiency, accessibility, financial-protection, cost-effectiveness, affordability | Y |
| [113] | E. R. Smith et al., “Cost-Effectiveness of Rotavirus Vaccination in Bolivia from the State Perspective,” Vaccine, vol. 29, no. 38, pp. 6704–6711, 2011. | English | Cost-effectiveness of vaccination in Bolivia | Bolivia | Yes | Cost-effectiveness study | cost-effectiveness | N |
| [114] | R. D. L. Ríos, C. Arósquipa, and W. Vigil-Oliver, “El financiamiento internacional para la cooperación al desarrollo de la salud de América Latina y el Caribe,” Rev. Panam. Salud Publica, vol. 30, no. 2, pp. 133–143, 2011. | Spanish | Donations (international cooperation) and health financing in LA | Latin America | Yes | Cross-sectional | efficacy, MDG | N |
| [115] | A. Mendes, M. G. Leite, and R. M. Marques, “Discutindo uma Metodologia para a Alocação Equitativa de Recursos Federais para o Sistema Único de Saúde,” Saude E Soc., vol. 20, no. 3, pp. 673–690, 2011. | Portuguese | Resource allocation in the health system of Brazil | Latin America | No | Stakeholder input | universality, efficiency, accessibility, financial-protection, cost-effectiveness, affordability | Y |
| [116] | L. G. Queiroz and L. Giovanella, “Agenda regional da saúde no Mercosul: arquitetura e temas,” Rev. Panam. Salud Publica, vol. 30, no. 2, pp. 182–188, 2011. | Portuguese | Health agenda for Mercosur countries | Argentina, Brazil, Paraguay, Uruguay, Venezuela | No | Government strategic plan for the health sector | equity, integration, mobility | N |
| [117] | A. Flores et al., “Advocacy and resource mobilization for rubella elimination in Guatemala,” J. Infect. Dis., vol. 204, no. Suppl 2, pp. S598-602, 2011. | English | Resource mobilization to vaccination in Guatemala | Guatemala | Yes | Economic evaluation  Mixed methods | cost-effectiveness | N |
| [118] | J. D. Kraemer, O. A. Cabrera, J. A. Singh, T. B. Depp, and L. O. Gostin, “Public health measures to control tuberculosis in low-income countries: ethics and human rights considerations,” Int J Tuberc Lung Dis, vol. 15, no. 6, pp. S19-24, 2011. | English | Ethics, human rights and control TB in LMICs | Latin America | No | Discussion paper | accessibility, reasonableness, effectiveness, proportionality, reciprocity, distributive-justice, trust, transparency | N |
| [119] | V. R. Leite, C. M. de Vasconcelos, and K. C. Lima, “Federalism and decentralization: impact on international and Brazilian health policies,” Int. J. Heal. Serv., vol. 41, no. 4, pp. 711–723, 2011. | English | Federalism and decentralization in the health system of Brazil | Brazil | No | Jurisdictional review | decentralization | N |
| [120] | C. Franco-Paredes, I. Hernández-Ramos, J. I. Santos-Preciado, and Grupo de Trabajo en Inmunizaciones del Sistema Mesoamericano de Salud Pública, “Inmunizaciones y equidad en el Plan Regional del Sistema Mesoamericano de Salud Pública,” Salud Publica Mex., vol. 53, no. Supl 3, pp. S323-332, 2011. | Spanish | Equity and immunization in Mesoamerica | Latin America | No | Stakeholder input | equity, social participation, protection of indigenous people, coverage, efficacy, cultural appropriateness, evidence-based | N |
| [121] | G. T. B. Araujo, J. E. Caporale, S. Stefani, D. Pinto, and A. Caso, “Is equity of access to health care achievable in Latin America?,” Value Heal., vol. 14, no. 5, pp. S8-12, 2011. | English | Equity in access to health in Latin America | Mexico, Argentina, Brazil, Colombia | No | Discussion paper | universality, equity, efficiency, transparency, accessibility, interculturality, financial-protection, evidence-based, market | Y |
| [122] | A. D. Bertoldi et al., “Household expenditures for medicines and the role of free medicines in the Brazilian public health system,” Am. J. Public Health, vol. 101, no. 5, pp. 916–921, 2011. | English | Household expenditure for medicines in Brazil | Brazil | Yes | Cross-sectional | Public financing | N |
| [123] | R. J. F. Esteves, “The quest for equity in Latin America: a comparative analysis of the health care reforms in Brazil and Colombia,” Int. J. Equity Health, vol. 11, pp. 1–6, 2012. | English | Equity in the health system reforms in Colombia and Brazil | Brazil, Colombia | Yes | Cross country comparison/Case Study | equity, efficiency, social participation, decentralization, integrality, accessibility, privatization, targeting | Y |
| [124] | A. C. G. do E. Santo, V. C. N. Fernando, and A. F. B. Bezerra, “Despesa pública municipal com saúde em Pernambuco, Brasil, de 2000 a 2007 Municipal,” Cienc. Saude ColetivaSaúde, vol. 17, no. 4, pp. 861–871, 2012. | Portuguese | Municipal public health spending in Pernambuco Brazil | Brazil | Yes | Cross-sectional | equality | N |
| [125] | F. Tobar, I. Drake, and E. Martich, “Alternativas para la dopcion de politicas centradas en el acceso a medicamentos,” Rev Panam Salud Publica, vol. 32, no. 6, pp. 457–463, 2012. | Spanish | Policies of drug access in Latin America | Latin America | No | Situation analysis | efficiency, sustainability, accessibility, affordability, privatization, market, competitiveness, enforcement of enforcement of regulation | Y |
| [126] | M. C. Pedroso and A. M. Malik, “Cadeia de valor da saúde: um modelo para o sistema de saúde brasileiro Healthcare,” Cien. Saude Colet., vol. 17, no. 10, pp. 2757–2772, 2012. | Portuguese | A model of health care management for Brazil | Brazil | No | Framework | management | N |
| [127] | C. W. Keck and G. A. Reed, “The curious case of Cuba,” Am. J. Public Health, vol. 102, no. 8, pp. e13-22, 2012. | English | Health system of Cuba | Cuba | No | Discussion paper | universality, efficiency, equality, decentralization, prevention, integration, effectiveness | Y |
| [128] | A. Stamford and M. Cavalcanti, “Legal decisions on access to medicines in Pernambuco, Northeastern Brazil,” Rev. Saude Publica, vol. 46, no. 5, pp. 791–799, 2012. | English | Legal decision on access to medicines in Brazil | Brazil | Yes | Cross-sectional  Mixed methods | universality, accessibility, comprehensiveness, free-access | N |
| [129] | A. Zúñiga Fajuri, “Un modelo de adjudicación de recursos sanitarios para Chile,” Acta Bioeth., vol. 18, no. 2, pp. 221–230, 2012. | Spanish | A model of health resource allocation in Chile | Brazil | No | Stakeholder input | universality, solidarity, social justice, equity, efficiency, equality, rationing | N |
| [130] | A. Uthoff, J. Miguel Sánchez, and R. Campusano, “The health insurance market: lessons on the conflict between equivalence and solidarity,” Cepal Rev., vol. 0, no. 108, pp. 141–159, 2012. | English | Equity, solidarity and equivalence in health system reform in Chile | Brazil | No | Situation analysis | universality, solidarity, equity, efficiency, accessibility, | N |
| [131] | J.-P. Alfred, “QUEL EST LE COÛT RÉEL DE LA COUVERTURE UNIVERSELLE EN SANTÉ EN HAÏTI ?,” Sante Publique (Paris)., vol. 24, no. 5, pp. 453–458, 2012. | French | Costs of Universal health coverage in Haiti | Haiti | No | Situation analysis | universality | N |
| [132] | B. Cabieses and M. Espinoza, “Redistributing health through public health policies in Latin America: fair to whom and fair how?,” Rev. Panam. Salud Publica, vol. 32, no. 5, pp. 387–388, 2012. | English | Fairness and redistributing of care in LA | Latin America | No | Commentary | social justice, equity, prevention | N |
| [133] | V. J. Wirtz, Y. Santa-Ana-Tellez, E. Servan-Mori, and L. Avila-Burgos, “Heterogeneous effects of health insurance on out-of-pocket expenditure on medicines in Mexico,” Value Heal., vol. 15, no. 5, pp. 593–603, 2012. | English | Effects of health insurance on out-of-pocket expenditure on medicines in Mexico | Mexico | Yes | Cross-sectional | equity, sustainability, accessibility, timely access, financial-protection | N |
| [134] | F. S. Vieira and P. Zucchi, “Financing of Pharmaceutical Services in Brazilian Public Health System,” Saude E Soc., vol. 22, no. 1, pp. 73–84, 2013. | English |  | Brazil | Yes | Cross-sectional | efficiency, sustainability, decentralization, availability, rationality | N |
| [135] | G. Paraje and F. Vásquez, “Health equity in an unequal country: the use of medical services in Chile.,” Int. J. Equity Health, vol. 11, no. 81, pp. 1–16, 2012. | English | Health equity in Chile | Chile | Yes | Cross-sectional | equality, equity, accessibility, solidarity, privatization | Y |
| [136] | A. Honda, “10 best resources on ... pay for performance in low- and middle-income countries,” Health Policy Plan., vol. 28, no. 5, pp. 454–457, 2013. | English | Pay-for-performance in LMICs | Latin America | No | Literature review | efficiency, quality, MDG | N |
| [137] | R. Bitran, “Explicit Health Guarantees for Chileans: The AUGE Benefits Package,” International Bank for Reconstruction and Development, Washington DC, 2013. | English | Social health insurance and universal coverage in Chile | Chile | No | Discussion paper | universality, equity, efficiency, sustainability, financial-protection, fiscal-sustainability | N |
| [138] | Grupo de Estudios de ética clínica de la sociedad médica de Santiago, “Dimensión ética en la organización de la atención de salud.,” Rev Med Chile, vol. 141, no. 6, pp. 780–786, 2013. | Spanish | Ethics in the management of health system | Chile | No | Discussion paper | social justice, efficiency, equality, sustainability, quality | N |
| [139] | M. Flynn, “Brazilian pharmaceutical diplomacy: social democratic principles versus soft power interests,” Int. J. Heal. Serv., vol. 43, no. 1, pp. 67–89, 2013. | English | Pharmaceutical international policy of Brazil | Brazil | No | Discussion paper | universality, solidarity, sustainability, accessibility, right | Y |
| [140] | F. Sánchez-Moreno, “La inequidad en salud afecta el desarrollo en el Perú,” Rev Peru Med Exp Salud Publica, vol. 30, no. 4, pp. 676–682, 2013. | Spanish | Equity in health in Peru | Peru | No | Discussion paper | universality, equity, gradualness, effectiveness, progressiveness | N |
| [141] | D. McIntyre, M. K. Ranson, B. K. Aulakh, and A. Honda, “Promoting universal financial protection: evidence from seven low- and middle-income countries on factors facilitating or hindering progress,” Heal. Res. Policy Syst., vol. 11, pp. 1–10, 2013. | English | Universal financial protection in health in LMICs | Costa Rica | Yes | Case study | universality, accessibility, financial-protection, universal-financial-protection | N |
| [142] | I. Rico-Alba and A. Figueras, “The fuzzy line between needs, coverage, and excess in the Mexican Formulary List: an example of qualitative market width analysis,” Eur J Clin Pharmacol, vol. 69, no. 4, pp. 949–956, 2013. | English | Coverage of health services in Mexico | Mexico | Yes | Cross-sectional | rationality, market, efficacy, safety | N |
| [143] | L. Morgan et al., “Financial incentives and maternal health: where do we go from here?,” J Heal. Popul Nutr, vol. 31, no. 4, pp. S8-22, 2013. | English | Financial incentives and maternal health in LMICs | Latin America | No | Literature review | sustainability, accountability, evidence, quality, utilization, cost-effectiveness | N |
| [144] | A. Zúñiga F, “Isapres, tribunal constitucional y distribución del derecho a cuidado sanitario,” Rev Med Chile, vol. 141, no. 4, pp. 514–518, 2013. | Spanish | Constitution right to health and health insurance in Chile | Chile | No | Situation analysis | universality, equity, efficiency, equality, decentralization, accessibility, timely access, right, quality, financing-coverage, sufficiency | Y |
| [145] | A. Glassman et al., “Impact of conditional cash transfers on maternal and newborn health,” J Heal. Popul Nutr, vol. 31, no. 4, pp. S48-66, 2013. | English | Conditional Cash transfer, MDG and maternal health in LMICs | Latin America | Yes | Quantitative systematic review | utilization, MDG | Y |
| [146] | S. Franco, “Entre los negocios y los derechos,” Rev Cub Salud Publica, vol. 39, no. 2, pp. 268–284, 2013. | Spanish | Ethic and private health insurance in Colombia | Colombia | No | Discussion paper | sustainability, market, right to health | N |
| [147] | M. Gragnolati, M. Lindelow, and B. Couttolenc, Twenty years of health system reform in Brazil: an assessment of the sistema unico de saude. Washington DC: World Bank, 2013. | English | Health system reform in Brazil | Brazil | No | Situation analysis | equity, efficiency, decentralization, accessibility, financial-protection, quality, accountability | Y |
| [148] | L. Reveiz et al., “Litigios por derecho a la salud en tres países de América Latina: revisión sistemática de la literatura,” Rev. Panam. Salud Publica, vol. 33, no. 3, pp. 213–222, 2013. | Spanish | Right to health litigation in Brazil, Colombia and Costa Rica | Costa Rica, Brazil. Colombia | Yes | Qualitative systematic review | accessibility, right to health, effectiveness | N |
| [149] | S. Leatherman, K. Geissler, B. Gray, and M. Gash, “Health financing: A new role for microfinance institutions?” 2013. | English | Health financing by microfinance institutions in four poor countries | Bolivia | Yes | Cross-sectional | financial-stability, affordability | N |
| [150] | R. M. Burke et al., “The burden of pediatric diarrhea: a cross-sectional study of incurred costs and perceptions of cost among Bolivian families,” BMC Public Health, vol. 13, no. 708, pp. 1–10, 2013. | English | Costs of pediatric diarrhea in Bolivia | Bolivia | Yes | Cross-sectional | financial-protection | N |
| [151] | N. Beyeler, A. York De La Cruz, and D. Montagu, “The impact of clinical social franchising on health services in low- and middle-income countries: a systematic review,” PLoS One, vol. 8, no. 4, p. e60669, 2013. | English | Clinical social franchising in LMICs | Latin America | Yes | Qualitative systematic review | equity, accessibility, quality, cost-effectiveness, utilization | N |
| [152] | J. Campbell et al., “Human resources for health and universal health coverage: fostering equity and effective coverage,” Bull World Heal. Organ, vol. 91, no. 11, pp. 853–863, 2013. | English | Equity, universal coverage and human resources | Mexico, Brazil | Yes | Case study | universality, social justice, equity, efficiency, sustainability, transparency, availability, acceptability, accessibility, quality, effectiveness, financial-protection, utilization, accountability | Y |
| [153] | M. K. Kim, R. J. Blendon, and J. M. Benson, “What is driving people’s dissatisfaction with their own health care in 17 Latin American countries?” Heal. Expect., vol. 16, no. 2, pp. 155–163, 2013. | English | Dissatisfaction with health care in LA | Latin America | Yes | Cross-sectional | universality, integrality, accountability, quality | Y |
| [154] | V. C. Bachelet, “Hospital concessions in Chile: where we are and where we are heading,” Medwave, vol. 14, no. 10, p. e6039, 2014. | Spanish | Hospitals concessions and private financing in Chile | Chile | No | Stakeholder position paper | market, privatization | Y |
| [155] | A. Mendes, “The public fund and the constraints of Brazilian universal health financing,” Saude E Soc., vol. 23, no. 4, pp. 1183–1197, 2014. | English | Universal health financing in Brazil | Brazil | No | Discussion paper | universality, private financing, public financing | N |
| [156] | M. Juan, “Hacia un Sistema Nacional de Salud Universal,” Cir Cir, vol. 82, no. 1, pp. 98–108, 2014. | Spanish | Universal health system in Mexico | Mexico | No | Government position paper | efficiency, timely access, quality, right to health, sufficiency, accountability | N |
| [157] | C. V. Machado, L. D. de Lima, and C. L. T. de Andrade, “Federal funding of health policy in Brazil: trends and challenges,” Cad Saúde Publica, vol. 30, no. 1, pp. 187–200, 2014. | English | Federal funding of health in Brazil | Brazil | Yes | Interrupted time series | universality, decentralization | N |
| [158] | A. Vargas-Bustamante and C. A. Méndez, “Health care privatization in Latin America: comparing divergent privatization approaches in Chile, Colombia, and Mexico.,” J. Health Polit. Policy Law, vol. 39, no. 4, pp. 841–86, Aug. 2014. | English | Health care privatization in LA | Mexico, Chile, Colombia | Yes | Comparative policy analysis | universality, decentralization, privatization, market | N |
| [159] | B. Cabieses and P. Bird, “Glossary of access to health care and related concepts for low- and middle-income countries (LMICs): a critical review of international literature,” Int. J. Heal. Serv., vol. 44, no. 4, pp. 845–861, 2014. | English | Access to health care in LMICs | Latin America | Yes | Quantitative systematic review | universality, equity, equality, availability, acceptability, accessibility, right to health, affordability, utilization | N |
| [160] | M. A. Espinoza and B. Cabieses, “Equidad en salud y evaluacion de tecnologias sanitarias en Chile,” Rev Med Chile, vol. 142, no. S1, pp. S45-49, 2014. | Spanish | Equity and health technology assessment in Chile | Chile | No | Discussion paper | equity, efficacy, effectiveness, cost-effectiveness | Y |
| [161] | C. Flood and A. Gross, “Litigating the right to health: what can we learn from a comparative law and health care systems approach.,” Heal. Hum. Rights, vol. 16, no. 2, pp. 62–72, 2014. | English | Litigating the right to health | Colombia, Brazil | Yes | Comparative policy analysis | equity, equality, right to health | N |
| [162] | J. D. Ament et al., “Health impact and economic analysis of NGO-supported neurosurgery in Bolivia,” J Neurosurg Spine, vol. 20, no. 4, pp. 436–442, 2014. | English | Health impact and economic analysis of NGO-supported neurosurgery in Bolivia | Bolivia | Yes | Cost-effectiveness study | solidarity, international-solidarity, cost-effectiveness | N |
| [163] | R. M. Burke et al., “The economic burden of pediatric gastroenteritis to Bolivian families: a cross-sectional study of correlates of catastrophic cost and overall cost burden,” BMC Public Health, vol. 14, no. 642, pp. 1–12, 2014. | English | Catastrophic costs of pediatric gastroenteritis in Bolivia | Bolivia | Yes | Cross-sectional | financial-protection | N |
| [164] | K. D. Rao, V. Petrosyan, E. C. Araujo, and D. McIntyre, “Progress towards universal health coverage in BRICS: translating economic growth into better health,” Bull. World Health Organ., vol. 92, no. 6, pp. 429–435, 2014. | English | Universal health coverage in BRICS | Brazil | No | Situation analysis | universality, equity, financial-protection, right to health | N |
| [165] | I. Garcia-Subirats et al., “Barriers in access to healthcare in countries with different health systems. A cross-sectional study in municipalities of central Colombia and north-eastern Brazil,” Soc. Sci. Med., vol. 106, pp. 204–213, 2014. | English | Access to health care in Brazil and Colombia | Brazil, Colombia | Yes | Cross-sectional | equity, efficiency, decentralization, accessibility, market, privatization | N |
| [166] | P. Rizo-Rios, A. G. Rivera, I. R. Oropeza, and O. C. Ramirez, “The Update of the Mexican Health Care Formulary and Supply Catalog in the Context of the Health Technology Assessment,” Value Heal. Reg. Issues, vol. 5, no. C, pp. 29–34, 2014. | English | Health benefits plan and health technology assessment in Mexico | Mexico | No | Government policy | efficiency, availability, effectiveness, efficacy, optimization, quality | Y |
| [167] | D. Class, E. Cavagnero, K. Ferl, and S. Rajkumar, “Costa Rica - Health financing profile,” International Bank for Reconstruction and Development, Washington DC, 2014. | English | Health financing profile of Costa Rica | Costa Rica | No | Jurisdictional review | universality, equity, sustainability | N |
| [168] | P. A. Mosquera et al., “Challenges of implementing a primary health care strategy in a context of a market-oriented health care system: the experience of Bogota, Colombia,” Int J Heal. Plann Mgmt, vol. 29, no. 4, pp. e347–e367, 2014. | English | Primary health care strategy in a context of a market-oriented system in Colombia | Colombia | Yes | Case study | efficiency, decentralization, market, primary-care | Y |
| [169] | G. Bloom, S. Henson, and D. H. Peters, “Innovation in enforcement of regulation of rapidly changing health markets,” Global. Health, vol. 10, no. 53, pp. 1–11, 2014. | English | Innovation in health markets in LMICs | Latin America | No | Literature review | availability, enforcement of enforcement of regulation, accountability, effectiveness, cost-effectiveness | N |
| [170] | P. Frenz, I. Delgado, J. S. Kaufman, and S. Harper, “Achieving effective universal health coverage with equity: evidence from Chile,” Health Policy Plan., vol. 29, no. 6, pp. 717–731, 2014. | English | Universal health coverage and equity in Chile | Chile | Yes | Cross-sectional | universality, solidarity, equity, effectiveness | Y |
| [171] | J. Frenk, “Leading the way towards universal health coverage: A call to action,” Lancet, vol. 385, no. 9975, pp. 1352–1358, 2015. | English | Universal health coverage and health system financing | Latin America | No | Discussion paper | universality, solidarity, equity, efficiency, social participation, transparency, right to health, effectiveness, quality, accountability, financial-protection | Y |
| [172] | M. A. Clark, “The Meanings of Universal Health Care in Latin America.,” J. Heal. Polit. Policy Law, vol. 40, no. 1, pp. 221–226, 2015. | English | The meanings of universal health care in Latin America | Costa Rica, Chile | No | Discussion paper | universality | Y |
| [173] | A. C. Laurell, “The Mexican Popular Health Insurance: Myths and Realities,” Int. J. Heal. Serv., vol. 45, no. 1, pp. 105–125, 2015. | English | Universal insurance coverage and health system reform in Mexico | Mexico | No | Discussion paper | universality, equity, sustainability, decentralization, financial-protection, quality, efficacy, private | N |
| [174] | D. Titelman, O. Cetrá­ngolo, and O. L. Acosta, “Universal health coverage in Latin American countries: how to improve solidarity-based schemes.,” Lancet, vol. 385, no. 9975, pp. 1359–1363, 2015. | English | Solidarity and universal health coverage in LA | Latin America | No | Discussion paper | universality, solidarity, equity, financial-protection | N |
| [175] | M. Bachelet, “Towards universal health coverage: applying a gender lens.,” Lancet, vol. 385, no. 9975, pp. e25-6, 2015. | English | Gender and universal health coverage in Chile | Chile | No | Commentary | cost-effective, gender-equity | Y |
| [176] | A. Castro, V. Savage, and H. Kaufman, “Assessing equitable care for Indigenous and Afrodescendant women in Latin America,” Rev Panam Salud Publica, vol. 38, no. 2, pp. 96–109, 2015. | English | Equity, gender, indigenous and health coverage in Latin America | Latin America | Yes | Qualitative Systematic review | equity, interculturality, indigeneity, afro descendant-equity, gender-equity | Y |
| [177] | P. E. Ekmekci and B. Arda, “Enhancing John Rawls’s Theory of Justice to Cover Health and Social Determinants of Health.,” Acta Bioeth., vol. 21, no. 2, pp. 227–236, 2015. | English | Rawlsian justice | Latin America | No | Discussion paper | justice, equity | Y |
| [178] | A. E. Yamin and A. Frisancho, “Human-rights-based approaches to health in Latin America.,” Lancet, vol. 385, no. 9975, pp. e26-9, 2015. | English | Human rights and health in LA | Latin America | No | Commentary | universality, equity, equality, social participation, transparency, right to health, accountability | N |
| [179] | J. Vega and P. Frenz, “Latin America: Priorities for universal health coverage,” The Lancet, vol. 385, no. 9975. pp. e31-32, 2015. | English | Universal health coverage in LA | Latin America | No | Commentary | universality, equity, efficiency, transparency, availability, acceptability, accessibility, prioritization, effectiveness, quality | Y |
| [180] | F. Pega, S. Y. Liu, S. Walter, and S. K. Lhachimi, “Unconditional cash transfers for assistance in humanitarian disasters: effect on use of health services and health outcomes in low- and middle-income countries,” Cochrane Database Syst. Rev., no. 9, p. 62, 2015. | English | Unconditional cash transfer for humanitarian crisis in LMICs | Latin America | Yes | Quantitative systematic review | effectiveness, evidence-based | N |
| [181] | R. Atun et al., “Health-system reform and universal health coverage in Latin America.,” Lancet, vol. 385, no. 9974, pp. 1230–1247, 2015. | English | Universal health coverage and health system reforms in Latin America | Costa Rica, Cuba, Mexico, Argentina, Brazil, Chile, Colombia, Peru, Uruguay, Venezuela | No | Framework | universality, solidarity, equity, efficiency, sustainability, decentralization, right, south-south-cooperation, financial-protection | Y |
| [182] | J. C. Kohler, N. Mitsakakis, F. Saadat, D. Byng, and M. G. Martinez, “Does Pharmaceutical Pricing Transparency Matter? Examining Brazil’s Public Procurement System,” Global. Health, vol. 11, pp. 1–13, 2015. | English | Pharmaceutical pricing in Brazil | Brazil | Yes | Cross-sectional | equity, efficiency, social participation, transparency, accessibility, effectiveness, accountability, responsiveness, inclusiveness | Y |
| [183] | D. Razzouk et al., “The impact of antipsychotic polytherapy costs in the public health care in Sao Paulo, Brazil,” PLoS One, vol. 10, no. 4, p. e0124791, 2015. | English | Costs of antipsychotic to the public health system in Brazil | Brazil | Yes | Cross-sectional | cost-effectiveness, evidence-based | N |
| [184] | M. C. Restrepo-Méndez et al., “Progress in reducing inequalities in reproductive, maternal, newborn, and child health in Latin America and the Caribbean: an unfinished agenda.,” Rev. Panam. Salud Publica, vol. 38, no. 1, pp. 9–16, 2015. | English | Inequalities in maternal health in LA | Latin America | Yes | Cross-sectional | equity, MDG | N |
| [185] | S. Leatherman and K. H. Geissler, “Providing primary health care through integrated microfinance and health services in Latin America.,” Soc. Sci. Med., vol. 132, pp. 30–37, 2015. | English | Primary health care and micro-finance in LA | Mexico, Nicaragua, Argentina, Bolivia, Peru | Yes | Case study | availability, acceptability, accessibility, affordability | N |
| [186] | V. Tangcharoensathien, A. Mills, and T. Palu, “Accelerating health equity: The key role of universal health coverage in the Sustainable Development Goals,” BMC Med., vol. 13, no. 101, pp. 1–5, 2015. | English | Universal health coverage, equity, MDG, SDG | Latin America | No | Discussion paper | universality, equity, sustainability, accessibility, MDG, primary-healthcare, financial-protection | Y |
| [187] | D. Cotlear et al., “Overcoming social segregation in health care in Latin America.,” Lancet, vol. 385, no. 9974, pp. 1248–1259, 2015. | English | Segregation and health care in Latin America | Latin America | No | Situation analysis | universality, free choice, decentralization, targeting, unification, right | Y |
| [188] | J. Grogger, T. Arnold, A. S. León, and A. Ome, “Heterogeneity in the effect of public health insurance on catastrophic out-of-pocket health expenditures: the case of Mexico,” Health Policy Plan., vol. 30, no. 5, pp. 593–599, 2015. | English | Public health insurance on catastrophic out-of-pocket health expenditures in Mexico | Mexico | Yes | Cross-sectional | financial-protection | N |
| [189] | A. Arredondo, E. Orozco, and R. Aviles, “Evidence on equity, governance and financing after health care reform: lessons for Latin American countries,” Saude E Soc., vol. 24, no. Supl 1, pp. 162–175, 2015. | English | Equity, governance and financing health system reform in Mexico | Mexico | Yes | Cross-sectional | equity, social participation, accountability, governance, financial-protection, targeting | Y |
| [190] | L. O. M. de Andrade et al., “Social determinants of health, universal health coverage, and sustainable development: case studies from Latin American countries.,” Lancet, vol. 385, no. 9975, pp. 1343–1351, 2015. | English | Universal health coverage in LA | Cuba, Brazil, Chile, Colombia | Yes | Case study | universality, equity, social participation, intersectorality | N |
| [191] | A. Thoumi, K. Udayakumar, E. Drobnick, A. Taylor, and M. McClellan, “Innovations In Diabetes Care Around the World: Case Studies Of Care Transformation Through Accountable Care Reforms,” Health Aff., vol. 34, no. 9, pp. 1489–1497, 2015. | English | Accountable health reforms and diabetes care in Mexico, USA and India | Mexico | Yes | Case study | accountability | N |
| [192] | N. Heredia, A. C. Laurell, O. Feo, J. Noronha, R. González-Guzmá­n, and M. Torres-Tovar, “The right to health: what model for Latin America?,” Lancet, vol. 385, no. 9975, pp. e34-7, 2015. | English | the right to health and universal health coverage in LA | Latin America | No | Commentary | universality, right to health | N |
| [193] | R. B. Saltman, “Health sector solidarity: A core European value but with broadly varying content,” Isr. J. Health Policy Res., vol. 4, no. 1, pp. 1–7, 2015. | English | Solidarity in Europe | NA | No | Discussion paper | solidarity | Y |
| [194] | C. E. Abadia-Barrero, “Neoliberal Justice and the Transformation of the Moral: The Privatization of the Right to Health Care in Colombia,” | English | The privatization of the right to health in Colombia | Colombia | Yes | Ethnographic study | Right to health, market, deservedness, justice | N |
| [195] | J. Mulligan, “Insurance Accounts: The Cultural Logics of Health Care Financing,” Med. Anthropol. Q., vol. 30, no. 1, pp. 37–61, 2016. | English | Insurance and health care financing Puerto Rico | Latin America | Yes | Ethnographic study | market, targeting, privatization | N |
| [196] | C. Hartmann, “Postneoliberal Public Health Care Reforms: Neoliberalism, Social Medicine, and Persistent Health Inequalities in Latin America.,” Am. J. Public Health, vol. 106, no. 12, pp. 2145–2151, 2016. | English | Health inequalities and public health care reforms in Latin America | Bolivia, Ecuador, Venezuela | No | Discussion paper | solidarity, efficiency, equality, free choice, decentralization, intersectorality, interculturality, protection of indigenous people, market, privatization, profit, cost-benefit, indigeneity | Y |
| [197] | C. M. Domingos, E. de F. de Almeida Nunes, B. G. Carvalho, and F. de F. Mendonça, “A legislação da atenção básica do Sistema Único de Saúde: uma análise documental,” Cad. Saude Publica, vol. 32, no. 3, p. e00181314, 2016. | Portuguese | Legislation in primary care in Brazil | Brazil | Yes | Documentary analysis | equity, social participation, integrality, accessibility | N |
| [198] | A. A. Portella and R. P. Teixeira, “Federalismo fiscal e efetividade da dignidade da pessoa humana: análise da posição do município na estrutura do financiamento público brasileiro e a escassez de recursos para as ações de saúde,” Rev. Direito Da Cid., vol. 8, no. 2, pp. 631–679, 2016. | Portuguese | Fiscal federalism, human dignity and financing of health system in Brazil | Brazil | No | Situation analysis | sustainability, right to health | N |
| [199] | H. Waitzkin and I. Hellander, “The History and Future of Neoliberal Health Reform: Obamacare and its predecessors,” Int. J. Heal. Serv., vol. 46, no. 4, pp. 747–766, 2016. | English | Affordable care act, health reforms in LA | Latin America | No | Discussion paper | market, right to health | N |
| [200] | M. Á. González-Block, A. Figueroa, I. García-Téllez, and J. Alarcón, “Asignación financiera en el Sistema de Protección Social en Salud de México: retos para la compra estratégica,” Salud Publica Mex., vol. 58, no. 5, pp. 522–532, 2016. | Spanish | Finance allocation in the Mexican health system | Mexico | Yes | Cross-sectional | universality, financial-protection, efficacy | N |
| [201] | K. G. Martinelli, E. T. dos Santos Neto, S. G. Nogueira da Gama, and A. E. Oliveira, “Access to prenatal care: inequalities in a region with high maternal mortality in southeastern Brazil,” Cien. Saude Colet., vol. 21, no. 5, pp. 1647–1657, 2016. | English | Access to prenatal care and inequalities in Brazil | Brazil | Yes | Cross-sectional | equity, availability, acceptability, accessibility | Y |
| [202] | M. R. Reich et al., “Moving towards universal health coverage: lessons from 11 country studies,” Lancet, vol. 387, no. 10020, pp. 811–816, 2016. | English | Universal health coverage and health system financing | Brazil, Peru | No | Situation analysis | universality | N |
| [203] | M. Matus-López, L. Prieto Toledo, and C. C. Pedraza, “Evaluació del espacio fiscal para la salud en Perú,” Rev Panam Salud Publica, vol. 40, no. 1, pp. 64–69, 2016. | Spanish | Fiscal opportunities to financing health in Peru | Peru | Yes | Cross-sectional | sustainability, accessibility | N |
| [204] | L. Avila-Burgos, L. Cahuan-Hurtado, J. Montañez-Hernández, E. Servan-Mori, B. Aracena-Genao, and A. del Río-Zolezzi, “Financing Maternal Health and Family Planning: Are We on the Right Track? Evidence from the Reproductive Health Subaccounts in Mexico, 2003-2012,” PLoS One, vol. 11, no. 1, p. e0147923, 2016. | English | Financing maternal health in Mexico | Mexico | Yes | Cohort study | efficiency, accessibility, financial-protection, accountability, MDG | Y |
| [205] | B. Hanson, KaraMcPake, “Managing the public-private mix to achieve universal health coverage,” Lancet, vol. 388, no. 10044, pp. 622–630, 2016. | English | Public-private mix and universal health coverage | Latin America | No | Literature review | universality | N |
| [206] | F. N. Alvarez, M. Leys, H. E. Rivera Mérida, and G. Escalante Guzmán, “Primary health care research in Bolivia: systematic review and analysis,” Health Policy Plan., vol. 31, no. 1, pp. 114–128, 2016. | English | Primary health care in Bolivia | Bolivia | Yes | Quantitative systematic review | universality, intersectorality, integrality, interculturality, primary-healthcare, efficacy, accountability | N |
| [207] | Defensoría del Pueblo, “Informe tutela y acceso a información-2016,” Bogotá, 2017. | Spanish | ‘Tutela” in Colombia | Colombia | No | Situation analysis | right to health | Y |
