# Supplementary material for: A framework for explaining the role of values in health policy decision-making in Latin America: a critical interpretive synthesis
Source: Health Res Policy Syst. 2020 Sep 7;18:100. doi: 10.1186/s12961-020-00584-y (PMC7487839; doi:10.1186/s12961-020-00584-y)
Supplement: Supplementary file 5 — Additional file 5. Values identified in papers reviewed. [file 12961_2020_584_MOESM5_ESM.docx]

**Supplementary material 5. Values identified in papers reviewed**

| Value | Number of papers addressing | Value | Number of papers addressing | Value | Number of papers addressing |
| --- | --- | --- | --- | --- | --- |
| Equity | 95 | Integration | 5 | Financial autonomy | 1 |
| Universality | 74 | Interculturality | 5 | Financial soundness | 1 |
| Efficiency | 72 | Intersectorality | 5 | Financial stability | 1 |
| Accessibility | 61 | Pluralism | 4 | Flexibility | 1 |
| Decentralization | 46 | Public financing | 4 | Free access | 1 |
| Quality | 38 | Redistribution | 4 | Gradualness | 1 |
| Financial protection | 35 | Sufficiency | 4 | Implementability | 1 |
| Right | 29 | Country solidarity | 3 | Inclusiveness | 1 |
| Sustainability | 28 | Cultural appropriateness | 3 | Individuality | 1 |
| Social participation | 27 | Demand subsidies | 3 | Justice | 1 |
| Solidarity | 27 | Institutional autonomy | 3 | Mobility | 1 |
| Privatization | 25 | Management | 3 | Multiculturalism | 1 |
| Accountability | 24 | Planning | 3 | Optimization | 1 |
| Effectiveness | 24 | Prioritization | 3 | Portability | 1 |
| Market | 21 | Profitability | 3 | Progressiveness | 1 |
| Equality | 18 | User satisfaction | 3 | Proportionality | 1 |
| Availability | 15 | Adjust | 2 | Protection vulnerable population | 1 |
| Targeting | 15 | Cost containment | 2 | Public participation | 1 |
| Efficacy | 13 | Democratization | 2 | Rationality | 1 |
| Millennium Development Goals (MDG) | 13 | Governance | 2 | Reasonableness | 1 |
| Affordability | 12 | Hierarchization | 2 | Reciprocity | 1 |
| Cost effectiveness | 12 | Professional autonomy | 2 | Regressiveness | 1 |
| Free choice | 12 | Responsiveness | 2 | Relevance | 1 |
| Primary healthcare (PHC) | 12 | Sovereignty | 2 | Safety | 1 |
| Rationing | 12 | Adequate use | 1 | Savings | 1 |
| Competitiveness | 11 | Afro descendent equity | 1 | Self financing | 1 |
| Social justice | 11 | Austerity | 1 | Self management | 1 |
| Utilization | 11 | Centralization | 1 | Separation of functions | 1 |
| Acceptability | 10 | Citizenship | 1 | Simplicity | 1 |
| Coverage | 10 | Community participation | 1 | Social cohesion | 1 |
| Transparency | 10 | Comprehensiveness | 1 | South-South cooperation | 1 |
| Integrality | 9 | Compulsoriness | 1 | Stewardship | 1 |
| Gender equity | 7 | Continuity | 1 | Suitability | 1 |
| Indigeneity | 6 | Cost benefit | 1 | Transferability | 1 |
| Prevention | 6 | Cost efficiency | 1 | Transparent procurement | 1 |
| Stewardship | 6 | Cultural autonomy | 1 | Trust | 1 |
| Timely access | 6 | Deservedness | 1 | Unification | 1 |
| Evidence based | 5 | Empowerment | 1 | Voluntariness | 1 |
| Fairness | 5 | Feasibility | 1 |  |  |
